# The effects of combined exercise training on glucose metabolism and inflammatory markers in sedentary adults: a systematic review and meta-analysis

**DOI:** 10.1038/s41598-024-51832-y

**Published:** 2024-01-22

**Authors:** Fernanda M. Silva, Pedro Duarte-Mendes, Ana M. Teixeira, Carlos M. Soares, José P. Ferreira

**Affiliations:** 1 University of Coimbra, Faculty of Sport Sciences and Physical Education, FCDEF, Coimbra, Portugal; 2 Research Unit for Sport and Physical Activity (CIDAF, Uid/Dtp/04213/2020), University of Coimbra, Coimbra, Portugal; 3 Department of Sports and Well-Being, Polytechnic Institute of Castelo Branco, Castelo Branco, Portugal; 4 Sport, Health and Exercise Research Unit (SHERU), Polytechnic Institute of Castelo Branco, Castelo Branco, Portugal; 5 Sport Physical activity and health Research & INnovation CenTer, SPRINT, Santarém, Portugal; 6 Molecular Physical-Chemistry R&D Unit, Department of Chemistry, University of Coimbra, Coimbra, Portugal

**Keywords:** Diagnostic markers, Predictive markers, Endocrine system and metabolic diseases, Biomarkers, Endocrinology, Risk factors, Diseases, Metabolic disorders

## Abstract

This systematic review and meta-analysis aimed to determine the magnitude of the effect of combined exercise training on glucose metabolism markers, adipokines, and inflammatory cytokines in non-diabetic sedentary adults. PubMed, Web of Science, Scopus, Cochrane Library electronic databases and reference lists of included studies were explored for randomized controlled trials (RCTs) that included physically inactive adults and provided combined training interventions (aerobic plus resistance exercise). Effects on fasting glucose and insulin, Homeostatic Model Assessment of Insulin Resistance (HOMA-IR), HbA1c, adiponectin, leptin, IL-6, TNF-α, and C-reactive protein (CRP) in exercise vs control groups were analyzed using random effects meta-analysis. The Cochrane Risk of Bias Tool for Randomized Trials 2.0 (RoB 2) was used to assess the risk of bias. A total of 24 RCTs were included in the quantitative analysis. Combined exercise training significantly decrease fasting glucose (standardized mean difference, SMD: − 0.474, 95% CI [− 0.829, − 0.120], *p* = 0.009, 35 study arms), fasting insulin (SMD: − 1.024, 95% CI [− 1.502, − 0.545], *p* < 0.001, 27 study arms), HOMA-IR (SMD: − 0.946, 95% CI [− 1.450, − 0.442], *p* < 0.001, 23 study arms), TNF-α (SMD: − 0.972, 95% CI [− 1.361, − 0.582], *p* < 0.001, 10 study arms), and CRP (SMD: − 0.507, 95% CI [− 0.818, − 0.196], *p* = 0.001, 14 study arms). No significant effects were observed for HbA1c, adiponectin, leptin, and IL-6 levels. Random effects meta-regression models by age, sex, and intervention length were not able to explain any of the variation in the effect size of HOMA-IR. Findings from this systematic review and meta-analysis suggest that combined exercise training improves some glucose metabolism markers and inflammatory parameters in sedentary adults without diabetes.

## Introduction

Physical inactivity represents a major public health problem and is considered a serious threat to body homeostasis by diverting physiologic metabolism toward harmful pathways^1^. Levels of physical inactivity in adults are particularly high worldwide, with a recent meta-analysis showing that only 17.2% of adults meet the aerobic and resistance physical activity recommendations^2^. Not only is physical inactivity highly prevalent, but sedentary behavior (any waking behavior with an energy expenditure of ≤ 1.5 METs while sitting, reclining, or lying^3^) continues to increase as well^4^.

Sedentary behavior and physical inactivity are now recognized as important causal factors in the development of insulin resistance (IR) and type 2 diabetes (T2DM)^1,4–6^. Evidence showed that even a short-term (14 days) reduction in physical activity with increased sedentary behavior leads to a reduction in multi-organ insulin sensitivity and muscle insulin sensitivity index, and concomitant increases in central and liver fat and LDL-cholesterol^5^. Although the mechanistic insight into these pathophysiological changes is not completely understood, there is considerable evidence suggesting that an inactive lifestyle can reduce insulin sensitivity by lowering energy expenditure, dysregulating lipid profile, and enhancing lipid storage^1,5^. Chronic low-grade systemic inflammation is also closely involved in the pathophysiology of IR^1^. Specifically, due to low energy expenditure, an inactive lifestyle provokes excess lipid accumulation surrounding vital organs in the abdomen (i.e., visceral fat mass), as well as within liver and muscle cells^7^. Adipose tissue is an endocrine organ that regulates glucose and lipid metabolism and secretes a wide variety of bioactive circulating mediators referred as adipokines, such as adiponectin and leptin, in addition to cytokines and chemokines such as tumor necrotic factor-α (TNF-α), interleukin-6 (IL-6), and monocyte chemoattractant protein 1 (MCP-1)^8,9^. These proteins have complex cross-talks with components of the immune system and can modulate their activity^1,9^. In this way, the accumulation of visceral/subcutaneous adipose tissue causes an increase in immune system activity and circulating levels of inflammatory cytokines, which has been shown to alter insulin signaling and considerably contribute to IR^1,9^. Importantly, IR plays a mechanistic role in the pathogenesis of numerous cardiovascular risks factors such as T2DM, metabolic syndrome, atherosclerosis, and hypertension^8,10^.

It is widely recognized that exercise has important public health benefits, representing a strong anti-inflammatory and metabolism-improving strategy^11^. Previous studies showed that aerobic training is an effective strategy for improving glucose metabolism outcomes^12–14^ and inflammatory markers^15,16^ in sedentary adults. Aerobic training plays an important role in reducing body fat by enhancing oxygen utilization^17^. Previous evidence reported that aerobic training improves muscle capillarity, which helps glucose diffuse from capillaries into muscle cells, changes the muscle fiber type, favoring substrate oxidation, and increases the quantity and size of mitochondria^18^. Furthermore, aerobic training increases the expression and/or activity of key signaling proteins involved in the regulation of glucose uptake and metabolism in skeletal muscle (such as GLUT4 translocation mediated by AMPK) and increases lipid turnover and/or oxidation^19,20^.

Previous interventions using resistance training have demonstrated that this mode of exercise can also improve markers of glucose metabolism^12,13^ and inflammation^21–23^ in sedentary adults through a different physiological mechanism from aerobic training^12,18^. The favorable effect of resistance training in glucose metabolism outcomes may be partially attributed to skeletal muscle hypertrophy, which increases the mass of tissue insulin-sensitive in terms of glucose storage and utilization^17,18^. Resistance training may also enhance insulin action stimulating the expression and activity of glucose transporters, insulin receptors, and other enzymes involved in glucose metabolism^18^. Importantly, the exercise training has important anti-inflammatory effects that may be mediated via both a reduction in visceral fat mass (with subsequent decreased released of adipokines), and the increased production and release of anti-inflammatory cytokines from contracting skeletal muscle (i.e., myokines)^11,24^. Higher levels of skeletal muscle strength and muscle mass are associated with lower circulating inflammatory markers^25^.

Numerous randomized controlled trials (RCT) have suggested that a combination of aerobic and resistance training is superior to aerobic or resistance training alone for improving insulin sensitivity, glycemic control, and inflammatory markers in several populations including obese adults with metabolic syndrome^14^, T2DM patients^26,27^, and obese adolescents^28^. According to Collins et al.^29^, these effects of combined training seem to be more than additive, suggesting that synergic mechanisms may be in play. Indeed, the current international guidelines recommends that adults engaged in 150 min/week of moderate-intensity or 75 min/week of vigorous aerobic physical activity and perform 2 or more sessions/week of resistance exercise to prevent glucose levels from deteriorating as well as manage glycemia within a normal range^30,31^. However, inconsistencies about the effects of combined aerobic and resistance training on glucose metabolism and inflammatory markers in sedentary adults without diabetes have been found in the literature. A study^32^ in obese middle-aged men reported that 24 weeks of combined moderate- to vigorous-intensity exercise training (aerobic plus resistance exercise) reduced the levels of circulating markers of subclinical inflammation such as leptin, C-reactive protein (CRP), increased adiponectin levels, and concomitantly improved homeostatic model assessment of IR (HOMA-IR) index. The study of Pérez-López et al.^33^ also found that 12 weeks of aerobic and resistance training in postmenopausal women reduced the circulating concentrations of cytokines implicated in glucose and lipid metabolism and reduced glycosylated hemoglobin A1c (HbA1c) and fat mass. Another study^14^ also observed that 12 weeks of combined training in obese adults with metabolic syndrome improved fasting glucose, insulin, HOMA-IR, and HbA1c. Further, Donges et al.^16^ observed that 12 weeks of combined training reduced TNF-α and IL-6 levels in sedentary overweight middle-aged men. However, other studies have failed to show significant effects of combined training on these outcomes. For example, the study of Amaro-Gahete et al.^34^ did not observe significant changes for HOMA-IR, CRP, and IL-6 after 12 weeks of a combined training program in obese men adults. Bonfante et al.^35^ applied a long-term combined training program of 24 weeks in sedentary obese men but did not find significant changes in HbA1c levels. Likewise, Álvarez et al.^36^ also did not find significant changes in glucose metabolism markers after 12 weeks of combined training in women with insulin resistance. Another two studies also failed to observe significant changes in inflammatory cytokines (i.e., IL-6 and CRP) after 16 weeks of combined training in obese male and female adults^37^ and middle-aged sedentary men^38^.

The beneficial effects of regular exercise training on both insulin sensitivity and inflammatory profile have been confirmed in previous systematic reviews and meta-analyses. A meta-analysis^39^ that included studies of adults showed that exercise programs significantly reduced levels of fasting insulin, HOMA-IR, and HbA1c, as well as the mediators of inflammation such as leptin, fibrinogen, and angiotensin II. They also observed that people aged < 50 years, men, and with T2DM, hypertension, dyslipidemia, or metabolic syndrome appeared to benefit more from exercise. Another meta-analysis^40^ also reported that exercise training programs were also effective in reducing HOMA-IR in patients with overweight or obesity, with T2DM or other comorbidities associated. However, these meta-analyses included studies with different types of exercise programs (i.e., aerobic training, resistance, HIIT, etc.), some of which were unsupervised and combined with diet interventions and included adults aged > 18 years with comorbidities associated, such as T2DM. Therefore, a specific focus on the effect of combined training alone (type of exercise recommended by health organizations) on glucose metabolism markers and inflammatory profile in previously sedentary adults without diabetes is lacking in previous reviews and meta-analyses. Thus, it becomes important to understand if combined exercise training is an effective strategy to improve these metabolic markers in this population.

Therefore, the main objective of this work was to undertake a systematic review and meta-analysis of RCTs to quantify the magnitude of the effect of combined exercise training on glucose metabolism markers (HOMA-IR and HbA1c), adipokines (leptin and adiponectin), and inflammatory cytokines (IL-6, TNF-α, CRP) in non-diabetic sedentary adults. The second objective was to provide more evidence to explore whether the magnitude of the effect in HOMA-IR index differed with exercise program duration and characteristics of the participants. We hypothesize that combined exercise training is an effective intervention to improve glucose metabolism and inflammatory markers in non-diabetic sedentary adults.

## Methods

This systematic review and meta-analysis followed the Preferred Reporting Items for Systematic Reviews and Meta-Analysis (PRISMA) guidelines^41^, the PERSiST guidance (PRISMA in Exercise, Rehabilitation, Sport medicine and SporTs science)^42^, and the recommendations of the Cochrane Handbook for Systematic Reviews of Interventions^43^. The study protocol was registered in the PROSPERO database (registration number CRD42023381237).

### Search strategy

Four electronic databases (PubMed, Web of Science, Scopus, and Cochrane Library) were searched for original studies published up to December 2022. Previous studies and reviews were screened to identify relevant keywords to include within each subject category. The literature search was performed according to the PICO strategy and included a mix of MeSH and free-text terms, as follows: (**P**) Population: physically inactive adults (aged between 18 and 64 years); (**I**) Intervention: combined exercise training (aerobic plus resistance exercise); (**C**) Comparator: non-exercise control; (**O**) Outcomes: glucose metabolism markers, adipokines, and inflammatory cytokine levels. The Boolean search terms were utilized (AND, OR, NOT). The detailed search strategy is shown in Supplementary Table S1. An advanced search by title was carried out in each database^44^. The reference lists of the selected studies were also checked to identify potential eligible articles.

### Study selection, inclusion, and exclusion criteria

Data from the search were imported into EndNote X20 (Thomson Researchsoft, EndNote 20.2.1 version) and all duplicates were removed. Titles, abstracts, and full texts were independently assessed for eligibility by two authors (FMS, CMS). Any disagreements were resolved by consensus or involving a third author (PD-M). The inclusion criteria were as follows: (1) The study must be an RCT written in English, Portuguese, Spanish or French; (2) The study subjects must be sedentary adults (18–64 years old) without regular physical activity (e.g., less than 150 min/week) before study enrollment; (3) The intervention group must have adopted a supervised/guided combined training program for at least 4 weeks^45^, whereas the non-exercise controls maintained their previous lifestyle (physical inactivity and the same nutritional pattern); (4) Outcomes measures included at least one of the main outcomes of glucose metabolism (i.e., fasting glucose, insulin, HOMA-IR, HbA1c), and/ or adipokines (i.e., leptin and/or adiponectin) and/or inflammatory markers (i.e., IL-6, TNF-α, CRP, IL-10, IL-1β, IL-15, IL-1ra); (5) The study must provide quantitative statistical information necessary to allow the calculation of effect sizes.

The exclusion criteria were as follows: (1) The subjects had severe chronic diseases (i.e., cardiovascular disease, cancer, Type 1 or T2DM or insulin-dependent, liver or kidney diseases, polycystic ovary syndrome), and/or physical or mental conditions that can compromise their capacity to exercise (i.e., limiting osteoarticular diseases, disability, frailty, Alzheimer’s disease or other), and/ or were in special conditions like women pregnant or on hormonal therapy, postpartum, or recent surgery; (2) Studies with exercise training on the aquatic environment; (3) Studies that combined exercise program with other health interventions, such as diet (caloric restriction), dietary supplements, cognitive training, or others; (4) Levels of circulating biomarkers are not directly measured; (5) The paper is a review article, editorial, commentary, observational or a case study. When the study provides insufficient data, authors were contacted to obtain the missing information. If no response was obtained, the article was excluded from the analysis. When more than one study referred to the same sample, we used the study that provided the most detailed data with the largest sample size.

### Data extraction

Two authors (FMS, CMS) independently extracted the data using standardized forms and discussed inconsistencies until consensus was obtained. The information extracted included the following: (i) Study information (authors, year of publication, country); (ii) Sample size in experimental and control groups; (iii) Participants’ demographic characteristics (i.e., sex, age, body mass index (BMI) for experimental and control groups); (iv) Characteristics of the intervention (mode, frequency, duration, and intensity) and comparison; (v) Percentage of adherence to the exercise program; (vi) Main assessed outcomes and results obtained for each group; and (vii) Other variables analyzed.

### Risk of bias of the individual studies

Two researchers (FMS and CMS) independently conducted a quality assessment, using the Cochrane Risk of Bias Tool for Randomized Trials 2.0 (RoB 2)^46^. Any disagreement was resolved through discussion or involving the third reviewer (PD-M). The RoB 2 covers bias in 5 bias domains, namely: randomization process, deviations from intended interventions, missing outcome data, measurement of the outcome, and selection of the reported result^46^. The authors answered one or more signaling questions within each domain. The available response options were “yes,” “probably yes,” “probably no,” “no,” and “no information.” These answers able judgments like “low risk of bias,” “some concerns,” or “high risk of bias”. An overall risk of bias judgment is obtained across the judgments in each domain.

### Statistical analysis and data synthesis

When at least 3 studies are available for an outcome, a meta-analysis was performed. For this reason, we were unable to pool data for interleukin-15 (IL-15), interleukin-1 beta (IL-1β), and interleukin-1 receptor antagonist (IL-1ra). Meta-analyses were separately conducted for each main outcome (i.e., glucose, insulin, HOMA-IR index, HbA1c%, adiponectin, leptin, IL-6, TNF-alpha, and CRP) using Comprehensive Meta-Analysis (CMA) Software version 4.0 (Biostat, Inc., Englewood, NJ USA). Meta-analyses were performed using random effects, assuming the heterogeneity among studies^43^. The effect measure used was the standardized mean difference (SMD) and 95% confidence interval (CI), as the included studies assessed the same outcome, but determine it in different ways^43^. The data extracted from the different studies for effect-size calculations included: a) the mean and standard deviation values of the outcome measurements in each group at baseline and follow-up, b) the number of participants for whom the outcome was assessed in each group, and c) the effects direction. The effect-sizes were interpreted according to Cohen’s specifications, i.e., values of 0.8, 0.5, and 0.2 for large, medium, and small SMD, respectively^47^. We performed a meta-analysis using forest plots and the statistical significance of the results was set as *p* ≤ 0.05. When studies reported standard error (SE) of the mean data instead of the SD, this value was converted to SD, by the formula suggested in the Cochrane Handbook^43^. Since the pre- to post-assessment correlation coefficients were not reported in the included studies, a conservative *r* value of 0.7 was assumed^48,49^. Sensitivity analysis using the ‘one-study-removed’ procedure was also conducted. When an RCT included a control group and more than one combined exercise group (i.e., exercise programs with distinct intensities and/or linear vs. non-linear training), we separately labeled each exercise group and divided the number of participants of the control group by the number of intervention arms (exercise groups). Furthermore, in RCTs that reported more than one follow-up measure, only those assessed immediately after the conclusion of the intervention were considered for the analysis. When the results were presented in the form of graphs/ figures, authors were contacted. In studies in which the authors did not comply with our request, the data presented in graphs/figures were extracted using the GetData Graph Digitizer software (Version 2.26.0.20, DR MyCommerce, Inc). Heterogeneity was tested using: (a) Q Cochran statistic, in which a *p*-value < 0.05 or a large Q statistic relative to its degree of freedom, represents evidence of heterogeneity in intervention effects^43^; (b) I-squared (*I*^2^) statistic (i.e., describes the percentage of the variability in effect estimates) that ranges from 0 to 100% and in which a value of 25%, 50% and 75% reflects low, moderate, and high observed heterogeneity, respectively^50^; (c) Tau-squared (τ^2^), that defines the variance of the true effect sizes between studies with a τ^2^ ≥ 1 representing substantial heterogeneity; and (d) τ statistic, that refers to the estimated standard deviation of underlying effects across studies^43^. The prediction interval from the random-effects meta-analysis was also calculated to present the extent of between-study variation^43,51^. Although prediction intervals are a popular way of expressing the amount of heterogeneity in a meta-analysis, they are founded on the assumption that the effects of the studies are distributed normally^43,51^. Prediction interval was calculated when we had at least ten or more studies, as recommended by Higgins et al.^43^.

Subgroup analyses were performed for HOMA-IR outcome to identify potential causes of heterogeneity among the studies. Characteristics of the participants, i.e., age (ages between 18- and 34-years old or ages between 35- and 64-years old), sex (women, men, or both), and length of intervention (≤ 12 or > 12 weeks) were predefined as potential sources of heterogeneity. Moreover, random effects meta-regression models were used to examine whether mean age of participants (ranging from 18.9 to 57.8 years old), sex (both sex, women, and men), and intervention length (ranging from 12 to 24 weeks) influenced heterogeneity. Meta-regression was not performed for inflammatory outcomes with significant heterogeneity because these indicators did not include at least ten or more studies^43^.

The publication bias was estimated through visual inspection of funnel plots, as well as using Egger’s intercept test^52^. The funnel plots are commonly used to assess the possibility that results are missing from a meta-analysis due to their magnitude or *p* value^43,53^. The Egger’s intercept test was used when at least 10 studies were included in this meta-analysis, due to the power of the test^43,53,54^.

## Results

### Study selection

**Figure 1:**
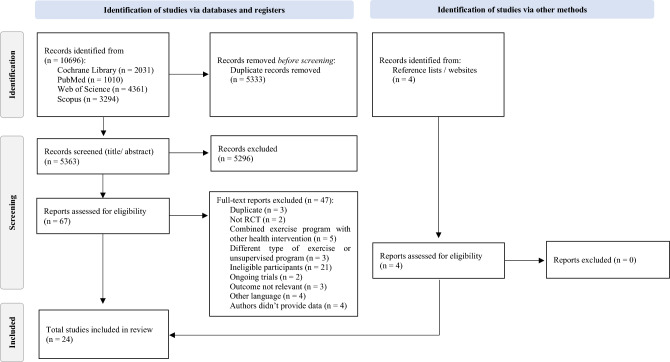
PRISMA flow diagram for the selection of studies. Adapted from: Page et al.^41^.

The systematic review flow diagram is presented in Fig. 1. We identified a total of 10,696 references through our electronic search. All references were checked for duplicates. After duplicate removal (n = 5333), a total of 5363 articles were initially screened by title and abstract. This number was reduced to 67 articles (we excluded n = 5296) that were subject to a full-text analysis, resulting in the additional exclusion of 47 articles. Twenty RCTs remained from the initial search, while 4 additional RCTs were manually added after checking reference lists and other sources. A total of 24 RCTs satisfied the inclusion criteria and were included in the present meta-analysis.

### Study characteristics

Characteristics and findings of the included studies are presented in Table 1. We included a total of 24 RCTs, published between 2005 and 2022. Studies were conducted in Europe (n = 7), South America (n = 8), Asia (n = 7), and Oceania (n = 2). As far as study design is concerned, 9 out of the 24 intervention protocols compared a combined training group vs. a non-exercise control group (i.e., 2-arm RCT). Additionally, 5 studies presented a 3-arm design, of which 2 included combined training programs with different training characteristics (i.e., moderate exercise vs vigorous exercise; non-periodized group vs periodized group). Also, 10 studies presented a 4-arm design, with different types of exercise.

**Table 1:** Characteristics of original studies.

Study	Participants							Intervention and comparasion	Outcomes			
Author, year, and country	Groups	N randomized [n (% female)]	N lost to follow-up [n (% female)]	N analyzed [*n* (% female)]	Age (years)	BMI (kg/m^2^)	Type of population	Exercise program	Adherence	Outcomes measures	Main results	Other variables
Silva et al. 2022^55^ Portugal	Exercise	22 (NA)	10 (NA)	12 (75)	46.58 ± 9.02	26.37 ± 3.93	Sedentary middle-aged workers (35–64 years old)	16 weeks, 3 sessions/week, 55 min/session	≥ 70%	Fasting glucose (mg/dL), fasting insulin (µm/L), HOMA-IR	↔ Glucose	Body composition, lipid profile, stress markers and quality of life
											↔ Insulin	
								Order: resistance exercise (RE) was followed by aerobic exercise (AE) in the same day (exercise session)			↔ HOMA-IR	
								RE: 25 min, 2 × 10–15 reps at 50–75% 1RM, 5–7 RPE; 7 exercises performed with free weights				
								AE: 15 min, fast walking/running/circuit exercises at 60–80% HRmax; RPE 6–8				
	Control	21 (NA)	2 (NA)	19 (78.9)	49.32 ± 7.13	28.75 ± 4.29		No exercise—maintaining their habitual lifestyle			↑ Glucose	
											↔ Insulin	
											↑ HOMA-IR	
Amaro-Gahete et al. 2021^34^ Spain	Exercise	6 (0)	0 (0)	6 (0)	41.3 ± 4.4	32.1 ± 3.6	Obese sedentary men (35–55 years old)	12 weeks, 3 sessions/week, 60 min/session	≥ 86%	Fasting glucose (mg/dL), fasting insulin (Ul/mL), HOMA-IR index, leptin (ng/mL), CRP (mg/dL)	For both groups:	Body composition, blood pressure, lipid profile, liver function, energy metabolism, echocardiography
								Order: Mixed circuit training methodology involving both exercises in the same day (exercise session)			↔ Glucose	
								Circuit training: 3–4 sets of 6–8 exercises			↔ Insulin	
								AE: treadmill and cycle-ergometer at 60–70% HRR			↔ HOMA-IR	
								RE: weight bearing, and free-weights exercises performed at 6–7 RPE			↔ Leptin	
											↔ CRP	
	Control	6 (0)	0 (0)	6 (0)	43.7 ± 6.1	32.5 ± 3.0		No exercise—maintaining their habitual lifestyle				
Amaro-Gahete et al. 2019^56^ Spain	Exercise	21 (NA)	4 (NA)	17 (52.9)	54.9 ± 4.5	25.41 ± 2.86	Sedentary middle-aged adults (40–65 years old)	12 weeks, 3 sessions/week, 210 min/week	≥ 90%	Fasting glucose (mg/dL), insulin (Ul/mL), HOMA-IR index	↔ Glucose	Body composition, blood pressure, lipid profile, liver function, cardiometabolic risk score
								Order: Not specified			↓ Insulin	
								RE: 60 min/week at 40–50% 1RM; exercises in weight-bearing and pneumatic machines			↓ HOMA-IR	
								AE: 150 min/week, 10 min bouts using treadmill, cycle-ergometer, and elliptical ergometer, at 60–65% HRR				
	Control	22 (NA)	5 (NA)	17 (58.8)	52.1 ± 4.1	26.67 ± 3.71		No exercise—maintaining their habitual lifestyle			↔ Glucose	
											↑Insulin	
											↑ HOMA-IR	
Brunelli et al. 2015^32^ Brazil	Exercise	27 (0)	10 (0)	17 (0)	49.29 ± 1.31	30.95 ± 0.40	Sedentary middle-aged obese men	24 weeks, 3 sessions/week, 60 min/session	≥ 85%	Fasting glucose (mg/dL), insulin (µUI/mL), HOMA-IR index, CRP (mg/L), IL-6, IL-15, TNF-α, IL-10 (pg/ml), Leptin (ng/mL), adiponectin (µg/mL)	↓ Glucose	Body composition, maximal-strength assessments
								Order: RE was followed by AE in the same day (exercise session)			↓ Insulin	
								RE: 3 sets × 6–10 RM, 6 exercises			↓ HOMA-IR	
								AE: 30 min, walking or running at 50–85% VO_2_peak			↔ IL-6	
											↔ TNF-α	
											↑ IL-15	
											↔ IL-10	
											↓ Leptin	
											↑ Adiponectin	
	Control	27 (0)	14 (0)	13 (0)	48.0 ± 1.72	31.01 ± 0.42		No exercise—maintaining their habitual lifestyle			↔ Glucose	
											↔ Insulin	
											↔ HOMA-IR	
											↔ IL-6	
											↑ TNF-α	
											↔ IL-15	
											↓ IL-10	
											↑ Leptin	
											↔ Adiponectin	
Bonfante et al. 2017^35^ Brazil	Exercise	27 (0)	15 (0)	12 (0)	49.1 ± 5.46	30.86 ± 1.52	Sedentary middle-aged obese men	24 weeks, 3 sessions/week, 60 min/session	≥ 85%	HbA1c%	For both groups: ↔ HbA1c (%)	Body composition, maximal-strength assessments, lipid profile, metabolic profile, FNDC5/irisin
								Order: RE was followed by AE in the same day (exercise session)				
								RE: 3 sets × 6–10 RM, 6 exercises				
								AE: 30 min, walking or running at 50–85% VO_2_peak				
	Control	27 (0)	17 (0)	10 (0)	49.1 ± 6.22	30.87 ± 1.79		No exercise—maintaining their habitual lifestyle				
Donges et al. 2013^16^ Australia	Exercise	13 (0)	0 (0)	13 (0)	46.2 ± 5.05	30.2 ± 2.52	Sedentary middle-aged men (40–65 years old)	12 weeks, 3 sessions/week, 60 min/session	≈ 92%	CRP, IL-6, TNF-α, IL-1ra	↔ CRP	Body composition, lipid profile, mRNA expression of GLUT4, peroxisome proliferator-activated receptor-ʸ coactivator-1α − β, etc
								Order: RE was followed by AE in the same day (exercise session)			↓ TNF-α	
								RE: 1.5–2 sets × 8–10 reps at 75–80% 1RM, 9 exercises			↓ IL-6	
								AE: 20–30 min, cycle ergometer with elliptical cross training at 75–80% HRmax			↔ IL-1ra	
	Control	8 (0)	0 (0)	8 (0)	49.5 ± 7.35	29.6 ± 5.94		No exercise—maintaining their habitual lifestyle			↔ CRP	
											↔ TNF-α	
											↔ IL-6	
											↔ IL-1ra	
Libardi etal. 2012^38^ Brazil	Exercise	11 (0)	0 (0)	11 (0)	48.5 ± 5.35	28.3 ± 3.0	Sedentary middle-aged healthy men	16 weeks, 3 sessions/week, 60 min/session	≥ 85%	Fasting glucose (mg/dl), TNF-α (pg/mL), IL-6 (pg/mL), CRP (mg/L)	↔ Glucose	Maximal strength, cardiorespiratory fitness, anthropometry
								Order: RE was followed by AE in the same day (exercise session)			↔ TNF-α	
								RE: 30 min, 3 sets × 8–10 RM, 6 exercises			↔ IL-6	
								AE: 30 min, walking or running at 55–85% VO_2_peak			↔ CRP	
	Control	13 (0)	0 (0)	13 (0)	49.1 ± 5.78	24.6 ± 3.3		No exercise—maintaining their habitual lifestyle			↑ Glucose	
											↔ TNF-α	
											↔ IL-6	
											↔ CRP	
Rahimi et al. 2020^14^ Iran	Exercise	12 (0)	2 (0)	10 (0)	44.9 ± 4.2	35.2 ± 1.3	Sedentary obese men with metabolic syndrome	12 weeks, 3 sessions/week, 60 min/session	≥ 80%	Fasting glucose (mg/dL), fasting insulin (ulU/mL), HOMA-IR, HbA1c (%)	↓ Glucose	Body composition, blood pressure, lipid profile, preptin, Change in circulating undercarboxylated osteocalcin, high molecular weight-plasma adiponectin
								Order: RE and AE were carried out on separated days. In weeks 1, 3, 5, 7, 9 and 11 they performed AE twice a week and RE once a week, while in weeks 2, 4, 6, 8, 10 and 12 they performed RE twice a week and AE once a week			↓ Insulin	
								RE: 45 min, 2–3 sets × 8–20 reps at 40–80% 1RM; 7 exercise weight machines			↓ HOMA-IR	
								AE: 43 min, 4 × 4 min intervals at 90% of HR peak walking/running on a treadmill, with 3 min exercise at 70% HR peak between each interval			↓ HbA1c (%)	
	Control	11 (0)	1 (0)	10 (0)	46.4 ± 5.1	36.0 ± 1.3		No exercise – maintaining their habitual lifestyle			↔ Glucose	
											↔ Insulin	
											↔ HOMA-IR	
											↔ HbA1c (%)	
Streb et al. 2021^57^ Brazil	Non-periodized exercise	23 (60.9)	0 (0)	23 (60.9)	34.2 ± 6.7	33.7 ± 3.0	Sedentary obese adults (20–50 years old)	16 weeks, 3 sessions/week, 60 min/session	64%	Fasting glucose (mg/dL), fasting insulin (mU/L), HOMA-IR	↔ Glucose	Body composition
								Order: AE was followed by RE in the same day (exercise session)			↔ Insulin	
								AE: 30 min, walking or running at 50–59% HRR			↓ HOMA-IR	
								RE: 20 min, 2 sets × 10–12 RM				
	Linear periodized exercise	23 (60.9)	0 (0)	23 (60.9)	35.6 ± 7.4	33.5 ± 3.1		16 weeks, 3 sessions/week, 60 min/session	61%			
								AE: 30 min, walking or running at 40–69% HRR				
								RE: 20 min, 2 sets × 8–14 RM, 6 exercises with weight training equipment				
	Control	23 (60.9)	0 (0)	23 (60.9)	34.2 ± 7.6	33.2 ± 2.4		No exercise – maintaining their habitual lifestyle			↔ Glucose	
											↔ Insulin	
											↔ HOMA-IR	
Streb et al. 2022^37^ Brazil	Exercise	46 (NA)	25 (NA)	21 (NA)	Mean age of total sample:	Mean BMI of total sample:	Sedentary obese adults (20–50 years old)	16 weeks, 3 sessions/week, 60 min/session	Not reported	CRP, IL-6	For both groups:	Lipid profile
					37 ± 1.0 years old	33.0 ± 0.4 kg/m^2^		Order: AE was followed by RE in the same day (exercise session)			↔ IL-6	
								AE: 30 min, walking or running at 50–59% HRR			↔ CRP	
								RE: 20 min, 2 sets × 10–12 RM, 6 exercises				
	Control	23 (NA)	8 (NA)	15 (NA)				No exercise—maintaining their habitual lifestyle				
Sillanpää et al. 2009^69^ Filand	Exercise	18 (100)	0 (0)	18 (100)	48.9 ± 6.8	23.1 ± 2.5	Sedentary women (39–64 years old)	21 weeks, 4 sessions/week, 60–90 min/session	Not reported	Fasting glucose (mmol/l), fasting insulin (mIU/l)	↔ Glucose	Body composition, blood pressure, lipid profile
								Order: RE and AE were carried out on separate days. It was not specified the order of exercise sessions			↓ Insulin	
								AE: 1º training cycle: 30 min 2 times a week under the level of their aerobic threshold and 10 min above the aerobic threshold; 2º cycle: 45 min varying in intensity from below the aerobic threshold to above the anaerobic threshold and 60-min training sessions under the level of their aerobic threshold; 3º cycle: 60–90 min, included 90 min of cycling at a steady pace under the aerobic threshold and every other session 60 min of cycling with intensities varying from under the aerobic threshold to over the anaerobic threshold				
								RE: 3–4 sets × 6–20 reps at 40–90% 1RM, 8 exercises				
	Control	12 (100)	0 (0)	12 (100)	51.4 ± 7.8	23.2 ± 1.8		No exercise – maintaining their habitual lifestyle			↔ Glucose	
											↔ Insulin	
Pérez-López et al. 2021^33^ Spain	Exercise	NA	NA	13 (100)	58.7 ± 2.9	33.8 ± 5.3	Sedentary postmenopausal women (50–65 years old)	12 weeks, 3 sessions/week, 60 min/session	86%	Fasting glucose (mmol/l), fasting insulin (mIU/l), HbA1c, HOMA-IR, IL-6, CRP, IL-13, IL-15	↔ Glucose	Body composition, lipid profile, fibroblast growth factor 21
								Order: AE was followed by RE in the same day (exercise session)			↔ Insulin	
								AE: 20 min, treadmill, cycle ergometer and elliptical machine at 55–75% HRR			↓ HbA1c	
								RE: 40 min, 3 sets × 8–12 reps at 65% 1RM, 6 exercises			↔ HOMA-IR	
											↓ IL-6	
											↓ IL-15	
											↔ CRP	
	Control	NA	NA	12 (100)	56.9 ± 5.8	34.9 ± 6.4		No exercise—maintaining their habitual lifestyle			↔ Glucose	
											↔ Insulin	
											↔ HbA1c	
											↔ HOMA-IR	
											↔ IL-6	
											↔ IL-15	
											↔ CRP	
Shabani et al. 2019^68^ Iran	Exercise	12 (100)	0 (0)	12 (100)	54.83 ± 4.72	28.4 ± 1.13	Sedentary overweight or obese women (50–60 years old)	8 weeks, 3 sessions/week, 90 min/session	Not reported	Fasting glucose (mg/dL), CRP (mg/dL)	↔ Glucose	Anthropometry, blood pressure, lipid profile
								Order: RE was followed by AE in the same day (exercise session)			↓ CRP	
								RE: 35 min, exercises with free weights at 50–80% 1RM; 8 exercises				
								AE: 40 min, running on a treadmill or pedaling on a bicycle ergometer at 50–80% exercise target HR				
	Control	12 (100)	2 (100)	10 (100)	56.90 ± 4.93	28.6 ± 2.4		No exercise—maintaining their habitual lifestyle			↔ Glucose	
											↔ CRP	
Azarbayjani et al. 2014^59^ NewZeeland	Exercise	NA	NA	10 (0)	22.9 ± 1.66	23.3 ± 1	Young sedentary men	12 weeks, 3 sessions/week, 60 min/session	Not reported	Fasting glucose (mg/L), fasting insulin (uUI/ml), HOMA-IR	↔ Glucose	Body composition, blood pressure, lipid profile, physical performance
								Order: AE was followed by RE in the same day (exercise session)			↓ Insulin	
								AE: 20 min, treadmill exercise at 60–70% HRR			↓ HOMA-IR	
								RE: 2 sets × 10 reps at 70% 1RM; 10 exercises				
	Control	NA	NA	10 (0)	22.9 ± 1.66	24 ± 0.8		No exercise—maintaining their habitual lifestyle			↔ Glucose	
											↔ Insulin	
											↔ HOMA-IR	
Álvarez et al. 2021^36^ Chile	Exercise	15 (100)	5 (100)	10 (100)	43.0 ± 8.0	29.1 ± 3.0	Physically inactive adult women with IR (25–60 years old)	12 weeks, 3 sessions/week, 60 min/session	83%	Fasting glucose (mg/dL), fasting insulin (µIU/dl), HOMA-IR	For both groups:	Body composition
								Order: RE was followed by AE in the same day (exercise session)			↔ Glucose	
								RE: 40 min, 60 s of concentric/eccentric voluntary movements, and 60 s of recovery at 20–50% 1RM, 8–10 RPE; 5 exercises with dumbbells, free weights, and bars			↔ Insulin	
								AE: 20 min, 8–12 high intensity cycling intervals of 60 s, interspaced with 120 s of passive recovery, at 80–100% HRmax, 8–10 RPE. The rest period decreased progressively (120 s to 60 s in the final 12^th^ week)			↔ HOMA-IR	
	Control	15 (100)	2 (100)	13 (100)	40.0 ± 11	28.3 ± 3.6		No exercise – maintaining their habitual lifestyle				
Ha et al. 2015^67^ Korea	Exercise	10 (100)	0 (100)	9 (100)	21.44 ± 1.94	24.71 ± 2.50	Sedentary female students (20–26 years old)	12 weeks, 3 sessions/week, 80 min/session	Not reported	Fasting glucose (mg/dL), fasting insulin (IU/mL), HOMA-IR	↔ Glucose	Body composition, Lipid profile
								Order: AE was followed by RE in the same day (exercise session)			↓ Insulin	
								AE: 30–40 min, treadmill running at 60–80% HRR, 11–16 RPE			↓ HOMA-IR	
								RE: 20–30 min, 10–15 RM 60–80% HRR, 11–16 RPE; 12 exercises with hydraulic equipment				
	Control	10 (100)	0 (100)	9 (100)	21.00 ± 2.00	24.00 ± 1.60		No exercise – maintaining their habitual lifestyle			↔ Glucose	
											↔ Insulin	
											↔ HOMA-IR	
Park et al. 2015^66^ Korea	Exercise	10 (100)	0 (0)	10 (100)	57.20 ± 2.57	26.02 ± 1.55	Sedentary postmenopausal middle-aged women with abdominal obesity	12 weeks, 3 sessions/week, 90 min/session	Not reported	TNF-α (pg/mL)	↓TNF-α	Body composition, lipid profile, physical fitness, IgA, IgG, IgM, CD14, Endotoxin
								Order: AE was followed by RE in the same day (exercise session)				
								AE: 40 min, running at 40–75% HRR				
								RE: 30 min, 3 sets × 8–12 reps at 60–70% 1RM; 10 exercises				
	Control	10 (100)	0 (0)	10 (100)	57.20 ± 1.69	26.80 ± 1.09		No exercise—maintaining their habitual lifestyle			↔ TNF-α	
Mendez-Gutierrez et al. 2022^58^ Spain	Exercise vigorous-intensity group	110 (68)	NA	35	22.1 ± 2.2	24.7 ± 4.2	Young sedentary adults (18–25 years old)	24 weeks, 3–4 sessions/week, 230 min/week	Not reported	Adiponectin (mg/dL), Leptin (ug/L), and IL-6 (pg/mL)	For both groups:	Body composition, lipid profile, brown adipose tissue, physical fitness
								Order: RE and AE were carried out on separated days. It was not specified the order of exercise sessions			↔ Adiponectin	
								AE: 75–150 min/week at 60–80% HRR; cycle ergometer, treadmill, and elliptical ergometer			↔ Leptin	
								RE: 80 min/week at 50–70% of 1RM			↔ IL-6	
	Exercise moderate-intensity group		NA	37				24 weeks, 3–4 sessions/week, 230 min/week				
								AE: 75–150 min/week at 60% HRR; cycle ergometer, treadmill, and elliptical ergometer				
								RE: 80 min/week at 50% of 1RM				
	Control		NA	37				No exercise—maintaining their habitual lifestyle				
Asad et al. 2012^60^ Iran	Exercise	14 (0)	1 (0)	13 (0)	21.38 ± 2.6	28.64 ± 3.76	Sedentary healthy male college students	8 weeks, 3 times/week	Not reported	Adiponectin (µg/mL)	↔ Adiponectin	BMI, VO_2_max
								Order: RE and AE were carried out on separated days, but the RE was always done first				
								AE: 25–40 min, running at 65–85% HRmax				
								RE: 3 × 10–15 reps of weight training exercise with machines and free loads				
	Control	10 (0)	0 (0)	10 (0)	21.44 ± 1.13	29.26 ± 4.27		No exercise—maintaining their habitual lifestyle				
Gonçalves et al. 2021^61^ Brazil	Exercise	12 (0)	2 (0)	10 (0)	38.8 ± 2	25.1 ± 0.8	Sedentary healthy men (30–60 years old)	12 weeks, 3 sessions/week, 50 min/session	> 75%	Fasting glucose (mg/dL), fasting insulin (µU/L), HOMA-IR	For both groups: ↔ Glucose	Body composition, blood pressure, lipid profile
								Order: RE and AE were carried out on same day (exercise session), however the order of exercises was alternated each week			↔ Insulin	
								RE: 1–2 sets × 8–12 RM; 10 exercises			↔ HOMA-IR	
								AE: 15–25 min, treadmill, and stationary bike in a randomized order at 50–60% HRR				
	Control	10 (0)	2 (0)	8 (0)	41.8 ± 2	25.4 ± 0.8		No exercise—maintaining their habitual lifestyle				
Salamat et al. 2016^62^ Iran	Exercise	11 (0)	0 (0)	11 (0)	22.9 ± 3.34	29.28 ± 2.17	Sedentary overweight men	8 weeks, 3 sessions/week	Not reported	IL-6 (pg/ml), IL-1β (pg/ml) and TNF-α (pg/ml)	↓ IL-6	
								Order: RE and AE were carried out on separated days. Participants trained 3 session endurance and resistance in two weeks alternatively			↓ IL-1β	Body composition
								AE: 20–33 min, running at 45–80% HRR			↔ TNF-α	
								RE: 3 sets × 6–12 reps at 50–85% 1RM; 9 circuit resistance exercises.				
	Control	10 (0)	0 (0)	10 (0)	23.8 ± 4.11	27.88 ± 3.42		No exercise—maintaining their habitual lifestyle			↔ IL-6	
											↔ IL-1β	
											↔ TNF-α	
Hara et al. 2005^63^ Japan	Exercise	7 (0)	0 (0)	7 (0)	18.4 ± 0.5	29.9 ± 3.8	Young obese male subjects	22 weeks, RE: 2–3 times/week, AE: 3 times/week, 80–90 min	Not reported	Fasting glucose (mg/dL), fasting insulin (uU/ml), HOMA-IR, leptin (ng/ml), adiponectin. (ug/ml)	↓ Glucose	Body composition, lipid profile, VO_2_max
								Order: AE was followed by RE (it is not clear if was performed on same day or in separate days)			↔ Insulin	
								AE: 30 min, treadmills, or cycle ergometer at 40.8–54.8% VO_2_max			↔ HOMA-IR	
								RE: 50–60 min, 3 sets × 10 reps at 80% 1RM; 7 exercises			↔ Leptin	
											↔ Adiponectin	
	Control	7 (0)	0 (0)	7 (0)	19.4 ± 1.0	33.5 ± 5.6		No exercise – maintaining their habitual lifestyle			↔ Glucose	
											↔ Insulin	
											↔ HOMA-IR	
											↔ Leptin	
											↔ Adiponectin	
Rossi et al. 2017^64^ Brazil	Exercise	20 (100)	5 (100)	15 (100)	62.4 ± 5.4	–	Sedentary overweight or obese women (50–60 years old)	16 weeks, 3 sessions/week, 60 min/session	Not reported	Fasting glucose (mg/dL)	↔ Glucose	Body composition, blood pressure, lipid profile, plasminogen activator inhibitor-1, triacylglycerol
								Order: RE was followed by AE in the same day (exercise session)				
								RE: 30 min, 3 sets × 8–15 RM repetitions; 9 exercises				
								AE: 30 min, running track. The participants were instructed to cover the distance in the shortest possible time. The intensity of training was 100% of anaerobic threshold				
	Control	20 (100)	12 (100)	8 (100)	62.8 ± 5.9	–		No exercise – maintaining their habitual lifestyle				
Álvarez et al. 2019^65^ Spain	Exercise	20 (100)	6 (100)	14 (100)	43 ± 6	29.8 ± 3.9	Overweight/ obese women with prediabetes (30–59 years old)	20 weeks, 3 sessions/week, 60 min/session	≥ 70%	Fasting glucose (mg/dL)	↔ Glucose	Lipid profile, body composition, blood pressure, cardiorespiratory fitness
								Order: RE was followed by AE in the same exercise session				
								RE: 50 min, 10–20% 1RM (each exercise was executed following 1 min of work and 1 min of passive recovery); 8 exercises performed with dumbbells				
								AE: 30 min, walking/running at 70% HRmax				
	Control	20 (100)	6 (100)	14 (100)	40 ± 6	32.0 ± 5.8		No exercise—maintaining their habitual lifestyle				

AE, aerobic exercise; BMI, body mass index; CRP, C-reactive Protein; IgA, Immunoglobulin A; IgG, Immunoglobulin G; IgM, Immunoglobulin M; HbA1c, glycated hemoglobin A1c; HOMA-IR, Homeostatic Model Assessment for Insulin Resistance; IL-, Interleukin; HRmax, maximum heart rate; HRR, heart rate reserve; min, minutes; RE, resistance exercise; RM, repetition maximum; RPE, rate of perceived exertion; TNF-α, Tumor necrosis factor alpha; Vo_2_max, maximal oxygen consumption.

The 24 included studies had 852 sedentary participants, 476 (55.87%) participants in the intervention group, and 376 (44.13%) in the control group. The mean age and BMI of all participants were 41.71 ± 13.08 years old and 29.03 ± 3.59 kg/m^2^. Males and females were presented in 5 studies^37,55–58^. Eleven studies included only male^14,16,32,34,35,38,59–63^ and 8 studies included only female adults^33,36,64–69^. Two of the included studies reported include prediabetic adults^14,65^. Another study included women with IR^36^. The remaining studies did not specify the number of normoglycemic and/or prediabetic participants (if any).

Duration of exercise training programs ranged from 8 to 24 weeks, with a median duration of 12 weeks and a median of 3 sessions/week (range 3 to 6 sessions/week), with each session length a median of 60 min (range of 50 to 90 min). The aerobic component involved different modes, such as running, walking, cycling, or circuit exercises for periods of 15 to 90 min/session. Exercise intensity was defined as the percentage of maximal aerobic capacity (i.e., VO_2max_), maximum heart rate (HR_max_) or heart rate reserve (HRR). The intensities of aerobic training range between 40–80% HRR, 60–100% HR_max_, or 50–85% peak oxygen uptake (VO_2peak_). The VO_2peak_ refers to the highest value of oxygen consumption measured during a graded exercise test^70^. One study prescribed aerobic training at individualized VO_2_max (40.8–54.8% VO_2_max) because the target was the improvement of fat metabolism^63^. Another study^64^ determined the intensity of aerobic training at 100% of anaerobic threshold, determined by critical velocity protocol. The study of Sillanpää et al.^69^ established loading intervals varying in intensity from below the aerobic threshold to above the anaerobic threshold. Based on the presented data, resistance training was prescribed with a range of 1–4 sets of 6–20 repetitions for 5–10 exercises at 20–90% of one-repetition maximum (1RM).

Seventeen studies performed aerobic plus resistance training in the same session. Of these, 9 studies start the exercise session with resistance exercise followed by aerobic exercise^16,32,35,36,38,55,64,65,68^, and 6 studies started the exercise session with aerobic exercise followed by resistance exercise^33,37,57,59,66,67^. One study combined aerobic and resistance exercise in the same session, however, the order of exercises was alternated each week^61^ and another study involved a mixed circuit training methodology^34^. Another study^63^ reported that aerobic exercise was followed by resistance exercise, however, it is not clear if was performed on the same day or on separate days. Five studies performed resistance and aerobic training in separate sessions during the week^14,58,60,62,69^. The study of Amaro-Gahete et al.^56^ only reports the training volume per week, not mentioning how it was applied (i.e., in the same session or in different training sessions).

Furthermore, adverse events were only reported in 7 (29%) of the 24 RCTs included. These 7 studies mentioned that no adverse events were observed during the intervention. The control groups were characterized by no exercise prescription. Reported outcomes included fasting glucose (17 studies), fasting insulin (13 studies), HOMA-IR index (11 studies), HbA1c % (3 studies), adiponectin (4 studies), leptin (4 studies), IL-6 (7 studies), TNF-α (5 studies), CRP (7 studies). All RCTs reported measures recorded immediately after the intervention period.

As additional outcomes, we collected from the included studies, the pre-and post-intervention data of body composition variables and VO_2max_ of the participants of both groups (Supplementary Table S2). Of the 24 RCTs included in this review, only 18 presented pre- and post-intervention data for at least one body composition variable. The studies of Amaro-Gahete et al.^34^, Shabani et al.^68^, and Hara et al.^63^ observed that the exercise program was effective to reduce the BMI of the participants. Regarding waist circumference, some of the studies also found a significant reduction in the exercise group after the intervention^14,35,55^. Regarding fat mass, of the 13 studies with available data, 10 found a significant reduction of fat mass (in kilograms or percentage) after the combined exercise program^14,16,32–35,59,63,66,69^. No significant changes were observed for control groups in any of the studies. In relation to skeletal muscle mass, only 1 of the 7 studies that presented data, found a significant increase in muscle mass after the exercise program^14^. Finally, of the 10 RCTs that presented data for VO_2max_, 8 found a significant improvement after combined exercise program^14,32,35,38,59,63,66,68^.

### Quality assessment

Of the 24 included studies, 3 studies were classified as having “low risk of bias”, 18 as having “some concerns”, and 3 were considered as having “high risk of bias”. RCTs were deemed to have “some concerns” for risk of bias mainly due to a lack of information about the randomization/ allocation process, potential deviations from the intended interventions and/or no previous publication of a specific analysis plan. For RCTs categorized as having a “high risk of bias”, this was mainly due to missing data outcome and/or problems regarding the measurement of the outcome. The quality assessment details of each criterion are available in Supplementary Figure S1.

### Effects of combined exercise on glucose metabolism outcomes

#### Fasting glucose

**Figure 2:**
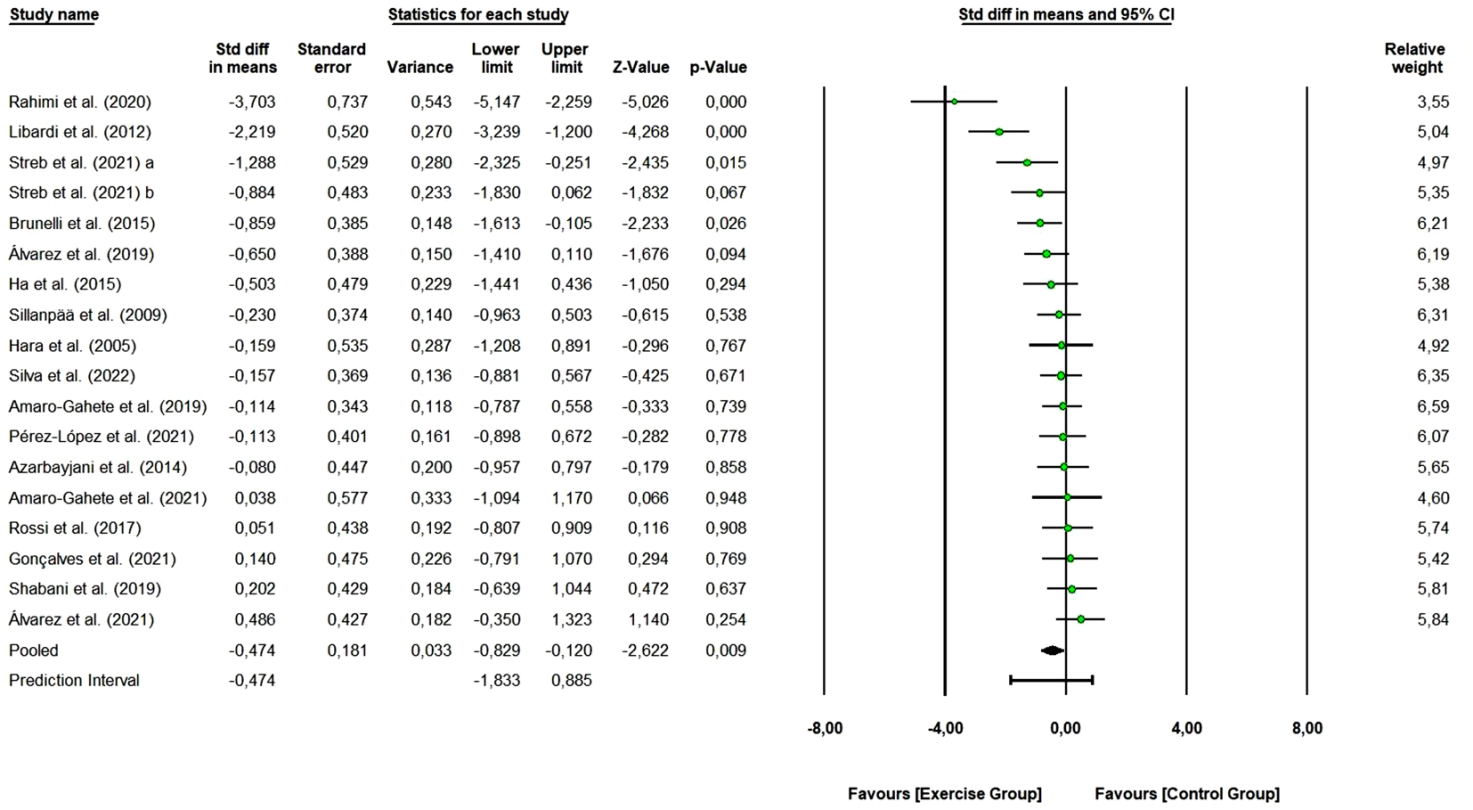
Pooled analysis for the effect of combined exercise training versus control on fasting glucose levels in physically inactive adults. The pooled SMD shows a reduction in fasting glucose levels in favor of the exercise group (combined training) compared to the control group. Streb et al. 2021 (**a**): non-periodized combined exercise vs. control group; Streb et al. 2021 (**b**): linear periodized combined exercise vs control group.

A total of 17 studies with 35 arms (411 participants, 195 in the control group and 216 in exercise group) were included to assess the overall effect of combined training program on fasting glucose levels. As shown in Fig. 2, the combined exercise reduced fasting glucose levels (SMD: − 0.474, 95% CI [− 0.829, − 0.120]; Z-value = − 2.622, *p* = 0.009) (small effect size). Of these, 15 studies were rated as “some concerns” or “low risk of bias”, and 2 studies as “high risk of bias”. Removing the “high risk of bias” studies did not change the overall effect of exercise (SMD: − 0.574 [95% CI − 0.949, -0.200], *p* < 0.003). One study removing sensitivity analyses did not change the overall result.

Regarding the homogeneity of the effects, a Q-value of 49.82 was obtained, with 17 degrees of freedom and a *p* < 0.001, indicating that we can reject the null hypothesis that the true effect size is the same in all the studies. The I^2^ was 65.88 (moderate heterogeneity), meaning that 66% of the variance in observed effects reflects variance in true effects. The τ^2^, variance of true effect sizes, had a value of 0.378 and τ statistic, the standard deviation of true effect sizes, was equal to 0.615. Assuming that the true effects are normally distributed (in d units), the estimated prediction interval is -1.833 to 0.885. We can observe that the true effect size in 95% of all comparable populations falls in this interval. The funnel plot (Supplementary Figure S2) shows that the distribution of the observed studies is not entirely symmetrical, suggesting that there was a possible publication bias or small sample effect. Egger’s test was also performed, and the intercept value was − 5.278 (95% CI [− 9.661, − 0.896], t-value = 2.55, df = 16). The recommended *p-value* (2-tailed) was 0.021.

#### Fasting insulin

**Figure 3:**
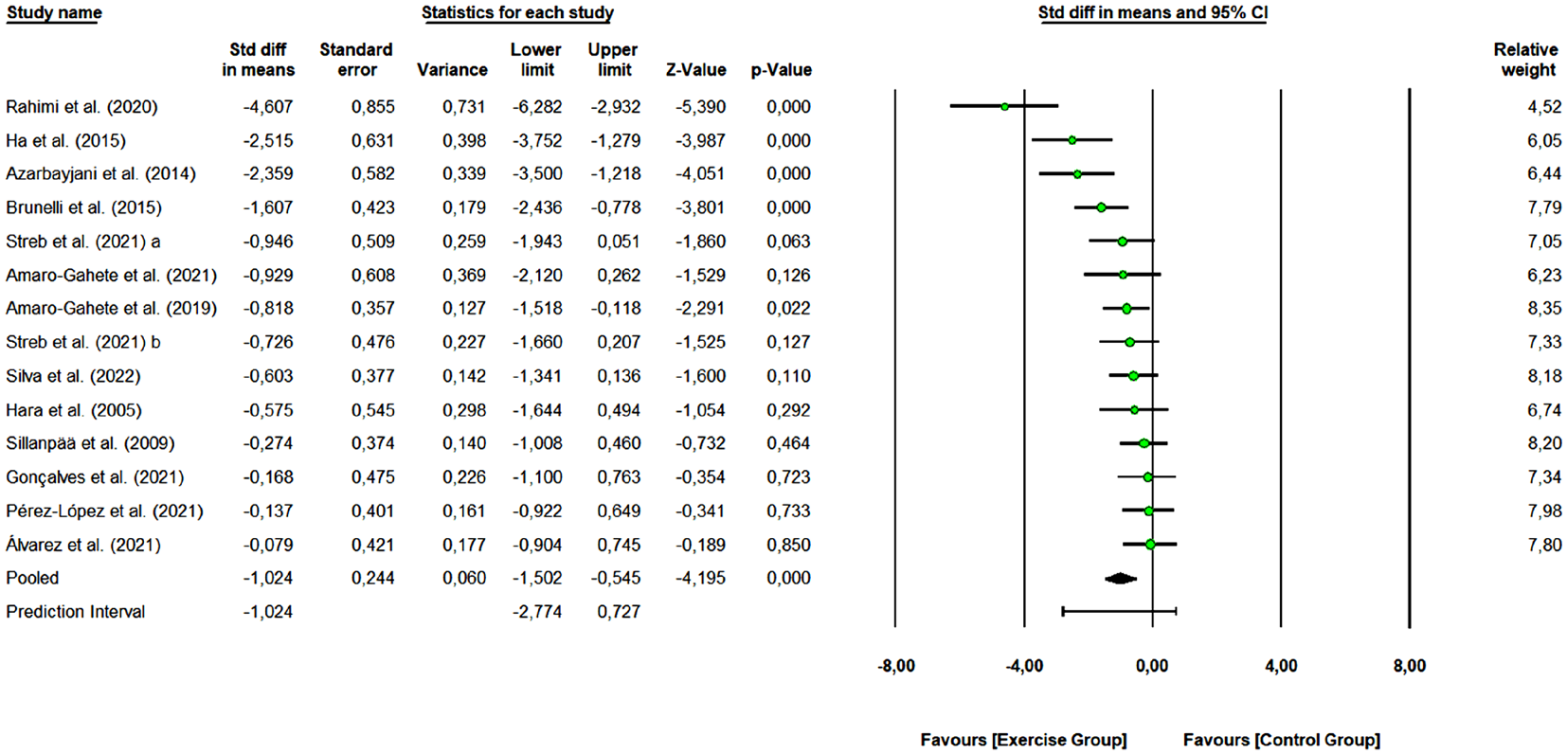
Pooled analysis for the effect of combined exercise training versus control on fasting insulin levels in physically inactive adults. The pooled SMD shows a reduction in fasting insulin in favor of the exercise group (combined training) compared to the control group. Streb et al. 2021 (**a**): non-periodized combined exercise vs control group; Streb et al. 2021 (**b**): linear periodized combined exercise vs control group.

A total of 13 studies with 27 arms (314 participants, 150 in the control group and 164 in exercise group) were included to assess the overall effect of combined training program on fasting insulin levels. Meta-analytic results are presented in Fig. 3. The combined exercise was effective in reducing the fasting insulin levels (SMD: − 1.024, 95% CI [− 1.502, − 0.545]; Z-value = − 4.195, *p* < 0.001) (large effect size). Eleven studies were rated as “some concerns” or “low risk of bias”, and 2 studies as “high risk of bias”. Removing the 2 “high risk of bias” studies did not change the overall effect of exercise (SMD: − 1.189 [95% CI − 1.720, − 0.659], *p* < 0.0001). One study removing sensitivity analyses did not change the overall result.

When verifying the homogeneity of the effects, a Q-value of 47.962 with 13 degrees of freedom and a *p* < 0.001 was obtained, which indicates that the true effect-size was not identical in all studies. We obtained a I^2^ of 72.895 (moderate heterogeneity), which tells us that some 73% of the variance in observed effects reflects variance in true effects. The τ^2^ was 0.586 and τ statistic 0.765. We can estimate that the prediction interval is − 2.774–0.727; we observed that the true effect size in 95% of all comparable populations falls in this interval. Regarding the publication bias, the funnel plot (Supplementary Figure S3) shows that the distribution of the observed studies is not entirely symmetrical. The Egger’s test shows an intercept value of − 6.198 (95% CI [− 9.96784, − 2.42906], t-value = 3.583, df = 12). The recommended *p-value* (2-tailed) was 0.004.

#### HOMA-IR index

**Figure 4:**
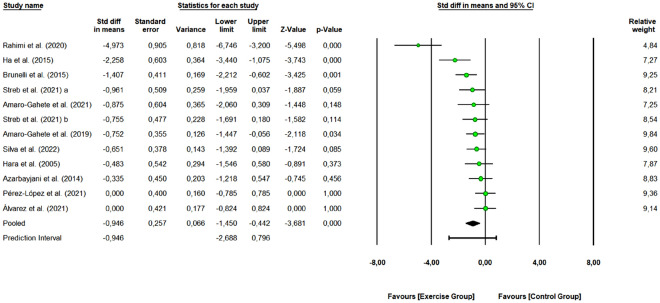
Pooled analysis for the effect of combined exercise training versus control on HOMA-IR index in physically inactive adults. The pooled SMD shows a reduction in the HOMA-IR index in favor of the exercise group (combined training) compared to the control group. Streb et al. 2021 (**a**): non-periodized combined exercise vs control group; Streb et al. 2021 (**b**): linear periodized combined exercise vs control group.

A total of 11 studies with 23 arms (266 participants, 130 in the control group and 136 in exercise group) were included to assess the overall effect of combined training program on IR (HOMA-IR index). The meta-analysis results presented in Fig. 4, demonstrated that combined exercise effectively reduced HOMA-IR index in the intervention group relative to that in the control group (SMD: − 0.946, 95% CI [− 1.450, − 0.442], Z-value = − 3.681, *p* < 0.001) (large effect size). Ten studies were rated as “some concerns” or “low risk of bias”, and 1 study as “high risk of bias”. Removing the “high risk of bias” study did not change the overall effect of exercise (SMD: − 1.042 [95% CI − 1.572, − 0.512], *p* < 0.0001). One study removing sensitivity analyses did not change the overall result.

The obtained Q-value was 38.6 with 11 degrees of freedom and a *p* < 0.001, so we can reject the null hypothesis that the true effect size is the same in all the studies. The I^2^ was 71.503 (moderate heterogeneity), i.e., 72% of the variance in observed effects reflects variance in true effects. The τ^2^ had a value of 0.545 and τ statistic was equal to 0.738. We can estimate that the prediction interval is − 2.688–0.796 and that the true effect size in 95% of all comparable populations falls in this interval. The funnel plot (Supplementary Figure S4) shows that the distribution of the observed studies is not entirely symmetrical. The Egger’s test resulted in an intercept value of − 5.743 (95% CI [− 9.928, − 1.558], t-value = 3.058, df = 10). The recommended *p-value* (2-tailed) was 0.012.

#### HbA1c (%)

**Figure 5:**
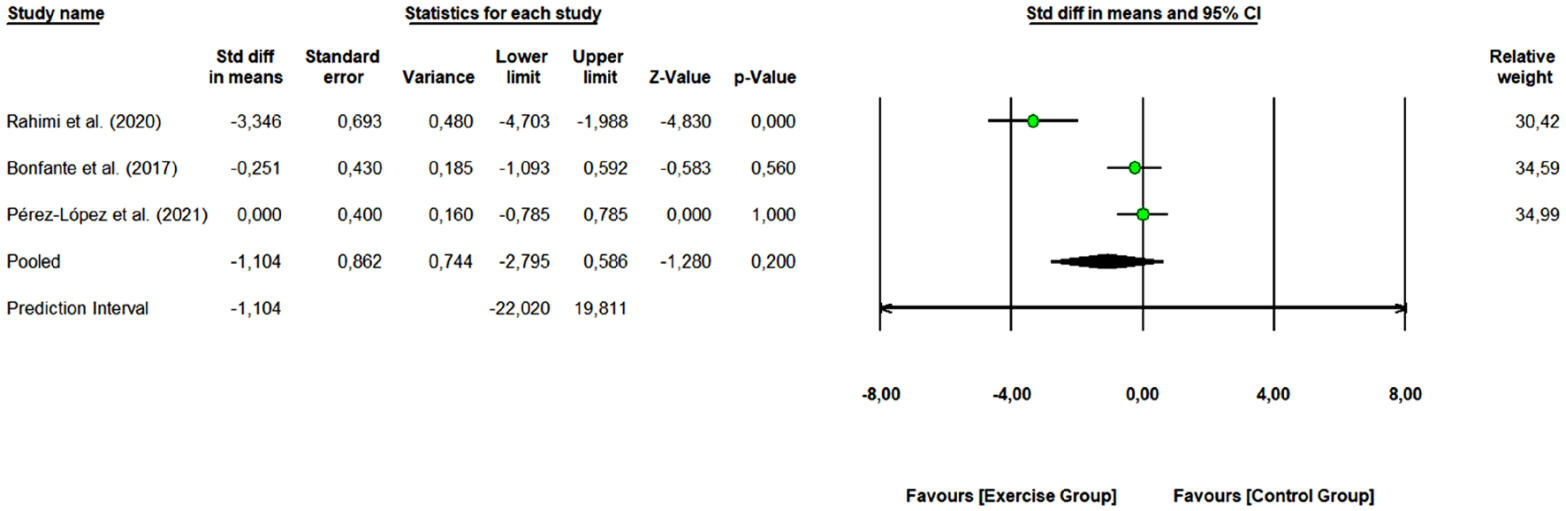
Meta-analysis for the effect of combined exercise training versus control on HbA1c levels (%) in physically inactive adults. The pooled SMD is not significant, however, a tendency to decrease HbA1c levels (%) is observed for the exercise group (combined training) compared to the control group.

A total of 3 studies with 6 arms (67 participants, 32 in the control group and 35 in exercise group) were included to assess the overall effect of combined exercise on HbA1c (%) levels. Pooled results (Fig. 5) revealed that combined exercise did not demonstrate a significant change in HbA1c levels (%) (SMD: − 1.104, 95% CI [− 2.795, 0.586], Z-value = -1.280, *p* = 0.200). The 3 studies were rated as “some concerns” or “low risk of bias”. One study removing sensitivity analyses did not change the overall result.

We obtained a Q-value of 18.616 with 2 degrees of freedom and *p* < 0.001, so we can reject the null hypothesis that the true effect size is the same in all the studies. The I^2^ was 89.257 (high heterogeneity), i.e., 89% of the variance in observed effects reflects variance in true effects. The τ^2^ had a value of 1.966 and τ statistic was 1.402.

### Effects of combined exercise on adipokines

#### Adiponectin

**Figure 6:**
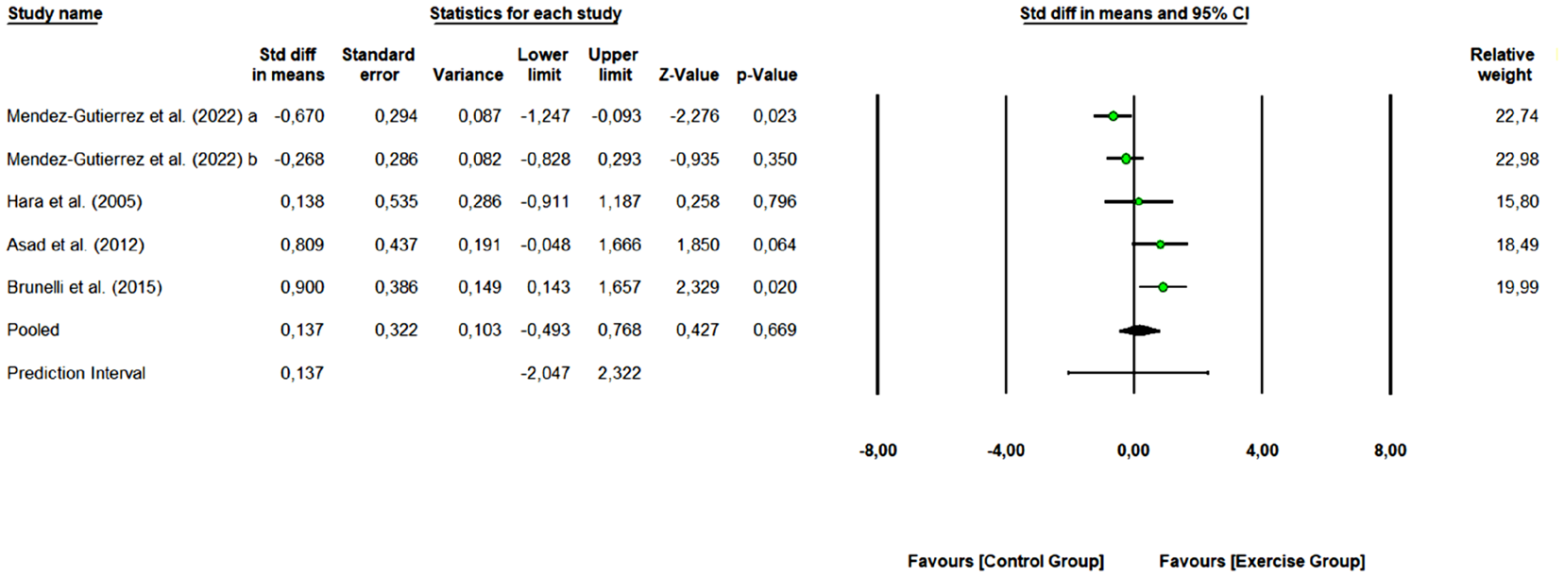
Meta-analysis for the effect of combined exercise training versus control on adiponectin concentrations in physically inactive adults. The pooled SMD is not significant, however, a tendency to increase adiponectin concentrations is observed for the exercise group (combined training) compared to the control group. Mendez-Gutierrez et al. 2022 (**a**): Moderate-intensity exercise vs control group; Mendez-Gutierrez et al. 2022 (**b**): Vigorous-intensity exercise vs control group.

A total of 4 studies with 9 arms (176 participants, 67 in the control group and 109 in exercise group) were included to assess the overall effect of combined training on adiponectin levels. Pooled results (Fig. 6) revealed that combined exercise did not demonstrate a significant change in adiponectin levels (SMD: 0.137, 95% CI [− 0.493, 0.768], Z-value = 0.427, *p* = 0.669). Of the 4 studies, 3 were rated as “some concerns” or “low risk of bias”, and 1 study as “high risk of bias”. Removing these “high risk of bias” study change the overall effect of exercise (SMD: 0.697 [95% CI 0.198, 1.196], *p* = 0.006) (moderate effect).

When verifying the homogeneity of the effects, a Q-value of 14.968 with 4 degrees of freedom and *p* = 0.005 was obtained, so we can reject the null hypothesis that the true effect size is the same in all the studies. The I^2^ was 73.277 (moderate heterogeneity), which tells us that some 73% of the variance in observed effects reflects variance in true effects. The τ^2^ was 0.368 and τ statistic 0.607.

#### Leptin

**Figure 7:**
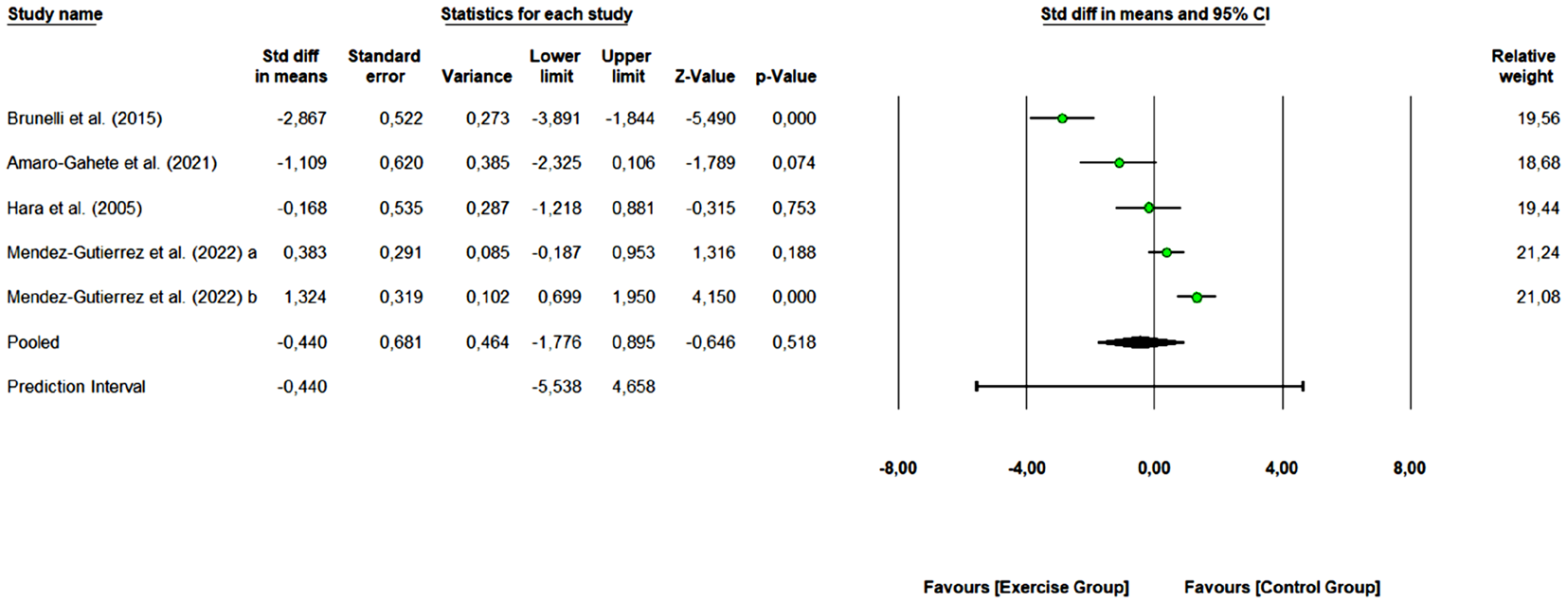
Pooled analysis for the effect of combined exercise training versus control on leptin concentrations in physically inactive adults. The pooled SMD is not significant, however, a tendency to decrease leptin concentrations is observed for the exercise group (combined training) compared to the control group. Mendez-Gutierrez et al. 2022 (**a**): Moderate-intensity exercise vs control group; Mendez-Gutierrez et al. 2022 (**b**): Vigorous-intensity exercise vs control group.

A total of 4 studies with 9 arms (162 participants, 62 in the control group and 100 in exercise group) were included to assess the overall effect of combined training on leptin levels. As shown in Fig. 7, combined exercise did not demonstrate a significant change in leptin levels (SMD: − 0.440, 95% CI [− 1.776, 0.895], Z-value = − 0.646, *p* = 0.518). Three studies were rated as “some concerns” or “low risk of bias”, and 1 study as “high risk of bias”. Removing the “high risk of bias” study did not change the overall effect of exercise (SMD: − 1.389 [95% CI − 3.023, 0.244], *p* = 0.095). One study removing sensitivity analyses did not change the overall result.

We obtained a Q-value of 51.992 with 4 degrees of freedom and *p* < 0.001, so we reject the null hypothesis. The I^2^ was 92.307 (high heterogeneity), i.e., 92% of the variance in observed effects reflects variance in true effects. The τ^2^ had a value of 2.102 and τ statistic was 1.450.

### Effects of combined exercise on inflammatory cytokines

#### IL-6

**Figure 8:**
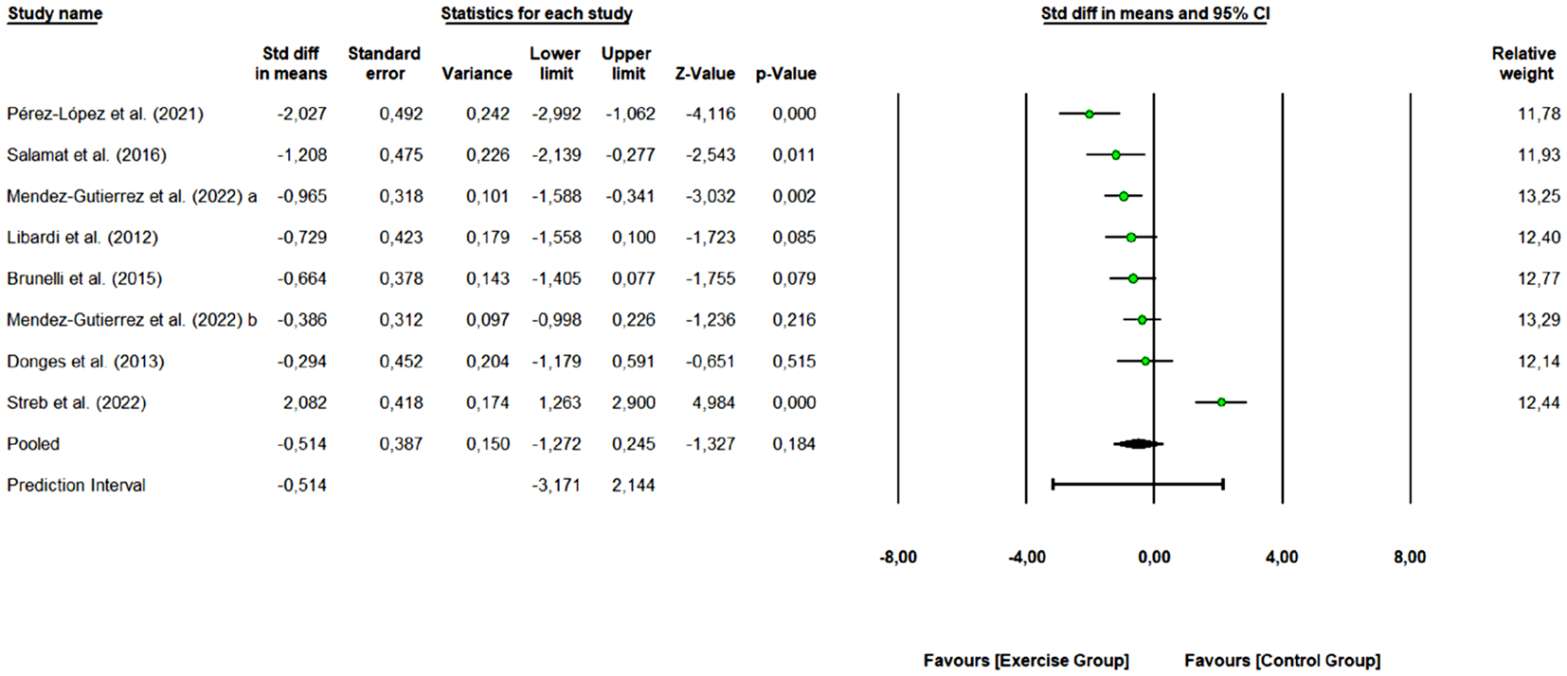
Pooled analysis for the effect of combined exercise training versus control on IL-6 levels in physically inactive adults. The pooled SMD is not significant, however, a tendency to decrease IL-6 levels is observed for the exercise group (combined training) compared to the control group. Mendez-Gutierrez et al. 2022 (**a**): Moderate-intensity exercise vs control group; Mendez-Gutierrez et al. 2022 (**b**): Vigorous-intensity exercise vs control group.

A total of 7 studies with 15 arms (253 participants, 103 in the control group and 150 in exercise group) were included to assess the overall effect of combined exercise on IL-6 levels. As shown in Fig. 8, combined exercise did not change IL-6 levels (SMD: − 0.514, 95% CI [− 1.272, 0.245], Z-value = − 1.327, *p* = 0.184). Six studies were rated as “some concerns” or “low risk of bias”, and 1 study as “high risk of bias”. Removing the “high risk of bias” study did not change the overall effect of exercise (SMD: − 0.462 [95% CI − 1.569, 0.645], *p* = 0.413). One study removing sensitivity analyses did not change the overall result.

We obtained a Q-value of 52.989 with 7 degrees of freedom and *p* < 0.001, so we reject the null hypothesis. The I^2^ was 86.79 (high heterogeneity), i.e., 87% of the variance in observed effects reflects variance in true effects. The τ^2^ had a value of 1.030 and τ statistic was 1.015.

#### TNF-α

**Figure 9:**
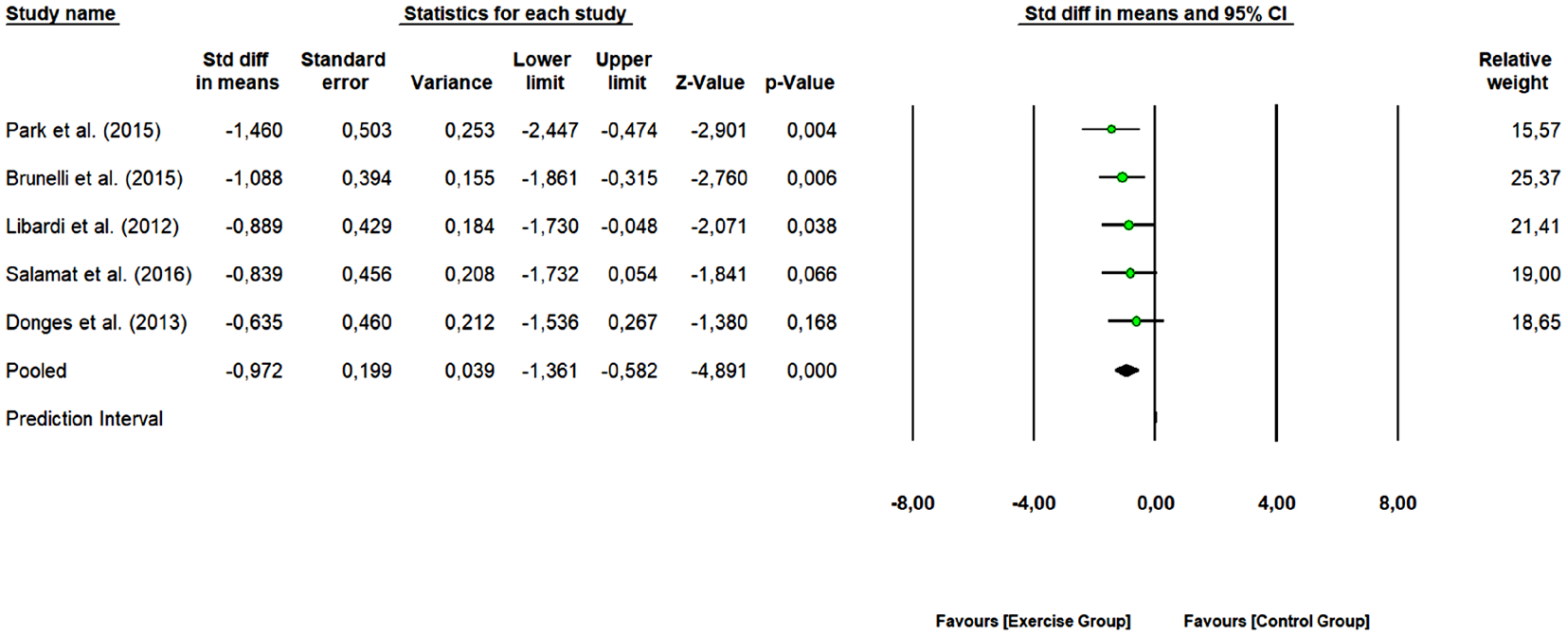
Pooled analysis for the effect of combined exercise training versus control on TNF-α levels in physically inactive adults. The pooled SMD shows a reduction in TNF-α levels in favor of the exercise group (combined training) compared to the control group.

A total of 5 studies with 10 arms (166 participants, 54 in the control group and 62 in exercise group) were included to assess the overall effect of combined training program on TNF-α levels. As shown in Fig. 9, combined exercise was effective in reducing TNF-α levels (SMD: − 0.972, 95% CI [− 1.361, − 0.582], Z-value = − 4.891, *p* < 0.001) (large effect size). The 5 studies were rated as “some concerns” or “low risk of bias”. One study removing sensitivity analyses did not change the overall result. The obtained Q-value was 1.689 with 4 degrees of freedom and *p* = 0.793. Therefore, the variance of true effects is estimated as zero, and all indices of heterogeneity (I^2^, τ^2^, and τ) are set to zero.

#### C-reactive protein

**Figure 10:**
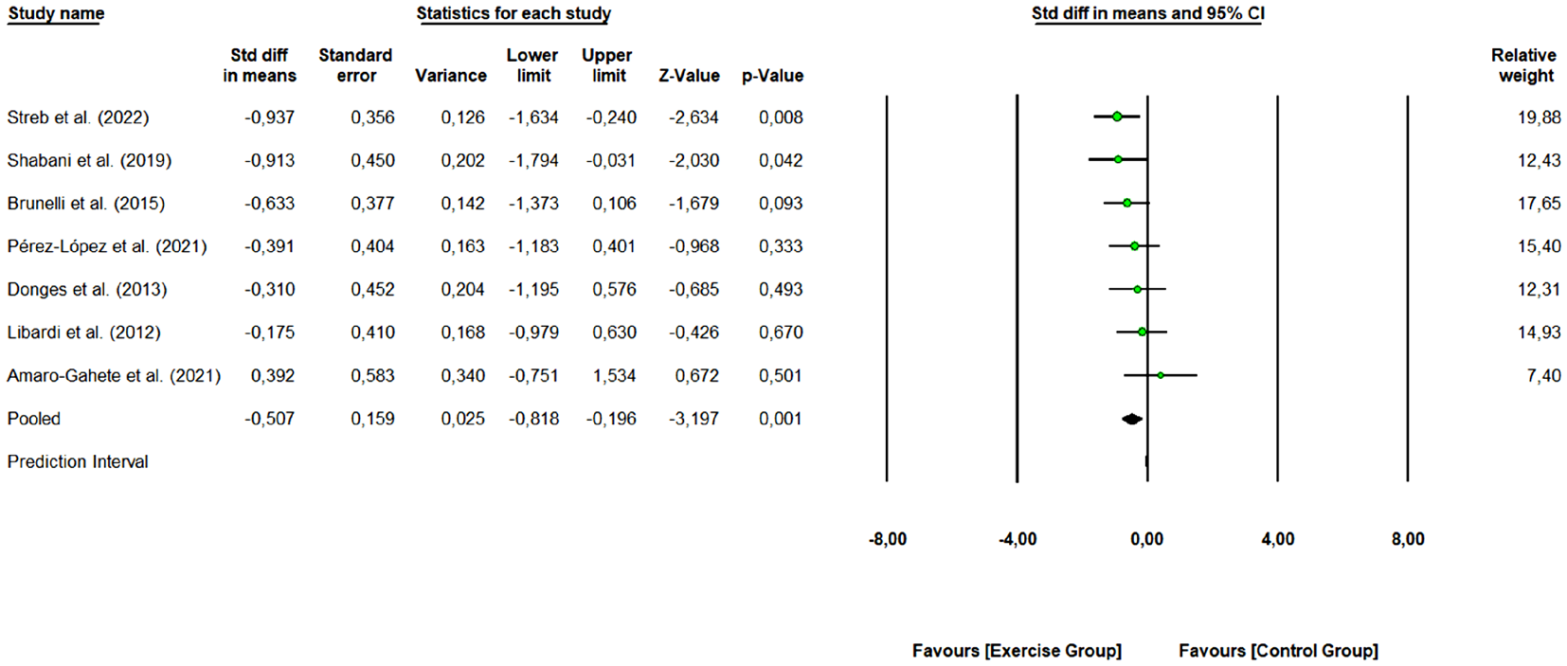
Pooled analysis for the effect of combined exercise training versus control on CRP levels in physically inactive adults. The pooled SMD shows a reduction in CRP levels in favor of the exercise group (combined training) compared to the control group.

Seven studies with 14 arms (170 participants, 77 in the control group and 93 in exercise group) were included to assess the overall effect of combined training program on CRP levels. As shown in Fig. 10, combined exercise was effective in reducing CRP levels (SMD: − 0.507, 95% CI [− 0.818, − 0.196], Z-value = − 3.197, *p* = 0.001) (medium effect size). The 7 studies were rated as “some concerns” or “low risk of bias”. One study removing sensitivity analyses did not change the overall result. The obtained Q-value was 5.693 with 6 degrees of freedom and *p* = 0.458. Therefore, the variance of true effects is estimated as zero, and all indices of heterogeneity (I^2^, τ^2^, and τ) are set to zero.

### Subgroup analysis and meta-regression

Subgroup analyses are presented in Table 2. When we classified studies based on sex (i.e., the inclusion of both sexes, women or men) analyses demonstrated a significant reduction in HOMA-IR index in studies with men (SMD = − 1.438, 95% CI [− 2.592, − 0.285], *p* = 0.015, I^2^ = 83%, 5 studies), and in studies with both sexes (SMD = − 0.757, 95% CI [− 1.164, − 0.350], *p* < 0.001, I^2^ = 0%, 3 studies) after exercise program. Studies with women participants did not demonstrate a significant change in HOMA-IR index (SMD = − 0.678, 95% CI [− 1.936, 0.581], *p* = 0.291, I^2^ = 82%, 3 studies). Moreover, when we classified studies based on subjects’ age, analyses demonstrated a significant reduction in HOMA-IR index after exercise program in studies with middle-aged adults (35–64 years old) (SMD = − 1.208, 95% CI [− 2.071, − 0.345], *p* = 0.006, I^2^ = 82%, 6 studies). The subgroup of studies with participants aged 18–34 years old presented no significant effect size (SMD = − 0.977, 95% CI [− 2.114, 0.160], I^2^ = 72%, 3 studies). Regarding to exercise program length, our analyses showed that both exercise interventions of ≤ 12 weeks (SMD = − 1.115, 95% CI [− 2.00, − 0.229], *p* = 0.014, I^2^ = 83%, 7 studies) and > 12 weeks (SMD = − 0.877, 95% CI [− 1.273, − 0.482], *p* < 0.001, I^2^ = 0%, 4 studies) of duration significantly reduced HOMA-IR index. The significant heterogeneity was not fully resolved by subgroup analysis. Moreover, a small number of studies qualifying for some subgroups is an important limitation to note. In this way, meta-regression analysis was performed to further clarify.

**Table 2:** Pooled estimates of HOMA-IR within different subgroups based on subjects’ age, and sex, as well as intervention length.

Group	No. of studies	No. of arms	No. of sample	SMD (95% CI)	*p value*	Q-value (*p-value)*	I^2^
Sex							
Men	5	10	96	− 1.438 (95% CI − 2.592, − 0.285)	**0.015**	23.205 (0.000)	83%
Women	3	6	66	− 0.678 (95% CI − 1.936, 0.581)	0.291	11.379 (0.003)	82%
Both	3	7	104	− 0.757 (95% CI − 1.164, − 0.350)	**0.000**	0.238 (0.971)	0%
Age*							
18–34 years	3	6	52	− 0.977 (95%CI − 2.114, 0.160)	0.092	7.209 (0.027)	72%
35–64 years	6	12	152	− 1.208 (95% CI − 2.071, − 0.345)	**0.006**	27.447 (0.000)	82%
Intervention Length							
≤ 12 weeks	7	14	152	− 1.115 (95% CI − 2.000, − 0.229)	**0.014**	35.568 (0.000)	83%
> 12 weeks	4	9	114	− 0.877 (95% CI − 1.273, − 0.482)	**0.000**	2.640 (0.620)	0%

Bold *p* values mean significant differences at *p* ≤ 0.05. * Streb et al. ^a, b^^57^ and Álvarez et al.^36^ were left out of the sub-analyses by age, as they included subjects aged 20–50 and 25–60 years, respectively.

Meta-regression model with sex as a potential explanatory variable resulted in a coefficient of − 0.567 (95% CI − 1.810 to 0.676) (*p* = 0.371) for studies with men and in a coefficient of 0.125 (95% CI − 1.258 to 1.508) (*p* = 0.859) for studies with women, compared to the reference subgroup “both sexes”. Thus, the set model by sex is not able to explain any of the variation in effect size. The meta-regression used to examine the relationship between age and effect size resulted in a coefficient of 0.0046 (95% CI − 0.038 to 0.047) (*p* = 0.8298). Therefore, this model is also not able to explain any variation in the effect size. Finally, the meta-regression model with intervention length as a potential explanatory variable resulted in a coefficient of 0.0033 (95% CI − 0.1248 to 0.1315) (*p* = 0.959). As age and sex, this model is not able to explain any variation in effect size. The detailed results of meta-regression models are presented in Supplementary Material.

## Discussion

This systematic review and meta-analysis provide a comprehensive analysis of the effects of the combined training program on glucose metabolism markers, adipokines, and inflammatory cytokines in non-diabetic sedentary adults. The quantitative analysis could only be performed for fasting glucose, insulin, HOMA-IR, HbA1c, adiponectin, leptin, IL-6, TNF-α, and CRP. Our hypothesis was not fully supported because the combined training did not significantly change HbA1c levels, adiponectin, leptin, and IL-6 concentrations. Importantly, the inflammatory markers results should be carefully interpreted due to the few studies included in the meta-analysis.

### Glucose metabolism markers

Our results showed that combined aerobic and resistance exercise programs were effective in reducing fasting glucose, fasting insulin, and IR index (measured by HOMA-IR) in sedentary adults without diabetes. However, no significant effect of combined training was observed for HbA1c %. Previous evidence showed that exercise enhances whole-body glucose uptake mainly by enhancing multi-organ insulin sensitivity and glucose disposal^71^. It is well established that exercise can improve glucose metabolism in the muscle, adipose, and hepatic tissues as well, leading to an overall increase in insulin sensitivity^72^.

These findings are in line with previous reviews that found a significant improvement in insulin sensitivity after an exercise program in overweight or obese adults with and without T2DM^40,73^. We also found that 6 of the RCTs included in this meta-analysis found a significant decrease in IR index after the combined exercise training^14,32,56,57,59,67^. These 6 RCTs involved sedentary adults with different clinical characteristics. Three studies included obese adults^32,57,67^, whereas 2 studies included sedentary adults with normal weight or overweight^56,59^. Another study^14^ included obese men with metabolic syndrome (including normoglycemic and prediabetic adults). Furthermore, the RCT studies included in this meta-analysis that did not observe significant decreases in HOMA-IR index after the combined training generally include overweight^55^ or obese adults^33,34,63^. Another study also included overweight women with IR^36^. The clinical characteristics of the participants are important, since Bird and Hawley^74^ reported that the potential to adapt and improve insulin sensitivity is likely to be influenced by the basal health state of the participants; healthy subjects, overweight/obese, and prediabetic metabolic syndrome are likely to respond to exercise differently in terms of adaptation and improvement.

These improvements in glucose metabolism markers may be explained by the synergistic effect of combined aerobic and resistance exercise training. Aerobic training has the potential to improve insulin sensitivity by increasing the expression and activity of many proteins involved in the regulation of glucose uptake and metabolism in skeletal muscle^18–20^. Furthermore, aerobic training may improve fat storage and fat oxidation capacity in muscles both in healthy and insulin-resistant individuals and increase capillary density which facilitates the diffusion of glucose from the capillaries into the muscle cells^18^. On the other hand, the beneficial effect of resistance training on insulin sensitivity may be explained in part by the hypertrophy of skeletal muscles^17,18,75^. An increase in muscle mass—induced by exercise training—may increase skeletal muscle glucose uptake and improve insulin sensitivity^75^. The greater skeletal muscle mass capillarity and its vasodilator response could explain this improvement^75^. In addition, resistance training also stimulates the expression and activity of glucose transporters, insulin receptors, and enzymes involved in glucose metabolism^18^.

It is well established that adipose tissue-induced inflammation is a cause of IR^1^. Increased visceral fat mass causes adipocyte dysfunction and adipokine dysregulation, which in turn stimulates inflammation by macrophage infiltration into adipose tissue^1,8^. This process results in both systemic and peripheral IR. In this way, previous evidence suggested that reducing visceral fat mass leads to the suppression of inflammation-induced IR and results in enhanced insulin sensitivity^76,77^. However, the literature is not consensual about this topic. Studies have reported that exercise training induced improvements in insulin sensitivity independently of weight and fat mass loss^12,78,79^, and that the benefits of exercise and weight loss are additive^80^. On the other hand, studies reported weight loss as the key component to improving insulin sensitivity in adults with metabolic syndrome^81,82^. In this meta-analysis, it was not feasible to determine the role played by combined exercise training in improving the HOMA-IR index, regardless of weight or fat mass loss. Nonetheless, most of the studies included in the analysis of HOMA-IR showed significant changes in body composition variables. A study^67^ observed that 12 weeks of combined training did not change body weight and muscle mass, but significantly reduced body fat (- 4.7%), insulin, and HOMA-IR in obese sedentary female adults, being effective in improving glucose metabolism. Similarly, the study of Azarbayjani et al.^59^ observed that 12 weeks of combined training promoted a significant reduction in body fat (- 2.1%) and waist-to-hip ratio, together with a reduction in fasting insulin levels and HOMA-IR in young sedentary men. According to the authors, the decrease in waist-to-height ratio and body fat together with the increased muscle strength of the lower and upper extremities may partly explain the enhancement in HOMA-IR. Brunelli et al.^32^ found that 24 weeks of moderate- to high-intensity combined training reduced markers of subclinical inflammation associated with obesity and improved HOMA-IR, and functional capabilities of obese middle-aged men. In relation to body composition, the exercise promoted a significant reduction of fat mass (-7.4%) and an increase of fat-free mass (+ 7.4%), without significant changes in body weight and BMI. The authors also found a significant association between CRP levels and the percentage of body fat reductions and remarked that the improvement in chronic inflammation after combined training is consequently associated with an improvement in insulin sensitivity. Amaro-Gahete et al.^56^ also found that 12 weeks of combined training significantly reduced BMI (− 0.51 kg/m^2^), waist circumference (− 1.90 cm), fasting insulin, and HOMA-IR. However, the authors did not find any association between changes in body composition variables and changes in HOMA-IR. The study by Rahimi et al.^14^ found a significant reduction in body weight, fat mass (%), waist circumference, and an increase in lean body mass (kg) as a result of 12 weeks of exercise training. Furthermore, a significant reduction in fasting glucose, insulin, HOMA-IR, and HbA1c were also observed. However, the authors did not explore the potential associations between the changes in body composition variables and changes in the HOMA-IR index, so the relation between weight loss and HOMA-IR changes remains inconclusive. On the other hand, a study^57^ observed that 16 weeks of non-periodized combined exercise training increased body weight (+ 1 kg) without changes in fat-free mass and body fat. Moreover, both non-periodized and periodized combined training similarly reduces IR markers in adults with obesity. According to the authors, it is possible that the muscular hypertrophy acquired with training (reported as an increase in maximum strength of upper and lower limbs) caused endocrine modulations, resulting in enhancement of insulin sensitivity, regardless of the change in body composition outcomes. Other studies included in our analysis showed that combined training promoted loss of body weight and fat mass, but was not effective in reducing fasting glucose, insulin, and HOMA-IR levels^33,34,63^. Future studies are needed to explore the role of weight and fat loss in the magnitude of glucose metabolism improvement in sedentary adults. The upregulation of insulin transports in the cellular membrane of insulin-dependent cells, improvement of β cell function, modulation of IRS-1 phosphorylation, and decreasing ceramide plasma levels, are other known potential molecular pathways by which aerobic exercise training improves insulin sensitivity^77^.

Our subgroup analysis by sex for HOMA-IR index showed a significant effect of exercise intervention in studies that included men or both sexes. No significant effect of exercise intervention was observed for the subgroup of studies that included women. However, due to the low number os studies included in each subgroup and the unresolved heterogeneity, a meta-regression analysis was performed to assess the relationship between sex and effect size, ignoring the potential impact of any confound. Our meta-regression model showed that sex (categorized as studies that include women, men, or both sexes) was not able to explain any variation in effect size. Additionally, when we performed a subgroup analysis based on subjects’ group age, we verified a significant reduction in HOMA-IR only in studies with middle-aged adults. These results appear to be in line with previous evidence that the risk of T2DM increases with age, being more prevalent in individuals aged 40–59 years when compared to younger individuals^83,84^. Specifically, with increasing age, insulin sensitivity, and the body’s glucose regulation ability gradually decreased^84^. However, based on the small number of studies included in subgroup analysis, a meta-regression model considering the mean age of the participants of each study was performed, however, no relationship was observed between mean age and effect-size. Lastly, subgroup analysis showed that both exercise interventions of ≤ 12 weeks and > 12 weeks of duration were effective in reducing HOMA-IR in sedentary adults. The meta-regression model for intervention length (number of weeks) as a potential explanatory variable showed that intervention length does not explain the variation in effect size. These results are interesting; however, it is important to note that the low number of studies included in most of the subgroup analysis, and the small sample sizes prevents us from establishing robust conclusions.

According to Bird and Hawley^74^ a dose effect may be evident, with greater exercise volumes and higher exercise intensities, producing greater benefit in insulin sensitivity. In our meta-analysis, we did not perform subgroup and/or meta-regression analysis with intensity and volume as potential explanatory variables of HOMA-IR effect, due to the high variability between the trials in report these training characteristics. The existing evidence about aerobic intensity and volume on these outcomes is somewhat conflicting. Studies that have examined a possible dose–response suggested that higher exercise doses (> 2000 kcal/week) are necessary to enhance insulin sensitivity and β-cell function in prediabetic adults^85^. Previous studies also suggested that higher-intensity aerobic exercise improves insulin sensitivity to a greater extent than low- or moderate-intensity exercise, while holding absolute exercise volume^86,87^. For example, DiPietro et al.^86^ evaluated older women in a 9-month aerobic training program with vigorous-intensity (80% of VO_2peak_), moderate-intensity (65% of VO_2peak_), and low-intensity control (50% of VO_2peak_), while holding constant prescribed exercise volume. They found that long-term vigorous-intensity aerobic training provides more benefits to insulin action compared with moderate- or low-intensity exercise. By contrast, a previous study^88^ reported that when controlled for total energy expenditure (i.e., exercise volume), moderate-intensity aerobic exercise improved insulin sensitivity more than vigorous-intensity aerobic exercise. According to the authors, one possible mechanism underlying this difference could be the enhanced metabolism of fatty acid stores in the skeletal muscle by moderate-intensity exercise, which may directly improve insulin sensitivity^88^. These findings were independent of weight loss or changes in body composition. Similarly, a recent RCT^89^ that conducted three combined aerobic and resistance training programs, with different intensities of aerobic training (moderate-, high- intensity training group, and alternated-intensity training group) for 12 weeks found that the insulin sensitivity (assessed by HOMA-IR and QUICKI indices) decreased in the three training groups, with greater magnitude of improvement observed by the moderate-intensity training group. Furthermore, the volume of resistance training needed to improve glucose metabolism markers remains uncertain, particularly when resistance training is combined with aerobic training^90^. A previous RCT^91^, evaluated the effects of different resistance training volumes (i.e., low-volume resistance training [3 sets/exercise] and high-volume resistance training [6 sets/exercise], with the same intensity, 70% 1RM) performed for 16 weeks, on muscular strength, and indicators of abdominal adiposity, metabolic risk, and inflammation in a cohort of post-menopausal women. The authors found that while low-volume resistance training improves HbA1c%, fat mass, and muscular strength, high-volume resistance training was able to improve indicators of abdominal adiposity, and lipid metabolism, and to prevent IL-6 increases. Another study^92^ that compared a low-intensity resistance training (without load increase) protocol with a high-intensity progressive resistance training protocol (i.e., 2–3 sets × 8 repetitions at 80% 1RM, 7 exercises) in a cohort of T2DM older adults, found that, in the high-intensity progressive training group, changes in skeletal muscle mass were significantly and inversely correlated with HOMA-IR and HbA1c. In contrast, in the low-intensity group, there was no such association, and the participants who increased muscle mass did not improve any glycemic control outcome^92^. More research is needed to explore the ideal intensity and volume of aerobic and resistance training that are needed to improve glucose metabolism outcomes, particularly when aerobic training is combined with resistance training.

### Adipokines and inflammatory cytokines

Our results suggest that combined exercise training programs were ineffective in improving adiponectin and leptin levels. However, when we removed the study with a high risk of bias from the adiponectin analysis, a significant positive effect of exercise was observed for adiponectin levels. These adipokines (adiponectin and leptin) and cytokines and chemokines (TNF-α, IL-6, CRP) are bioactive circulating mediators produced and released by adipose tissue^8,9^. Adiponectin is the most abundant protein secreted by adipose tissue and has strong anti-inflammatory properties^8^. Through the adiponectin receptors 1 and 2 (AdipoR1 and R2, respectively), adiponectin activates the AMPK and PPAR-α signaling pathways resulting in increased fatty acid oxidation and muscle glucose uptake together with suppressed gluconeogenesis in liver tissues^8^. Leptin reduces body fat by suppressing appetite and raising energy expenditure^93^. Dysregulated production or secretion of these adipokines has been directly associated with changes in IR through negative modulations in the sensitivity of insulin and leptin receptors^32,94^. This results consequently in changes in glucose uptake and transportation as well as hepatic glycogenesis, which have been linked to IR, and T2DM development^32,94,95^.

Brunelli et al.^32^ found that a 24-week combined exercise program was effective in reducing serum leptin, and in increasing serum adiponectin levels (+ 48%) in obese middle-aged men. According to the authors, increases in serum adiponectin concentrations observed in the exercise group may have influenced the improvements in IR parameters. However, other studies included in this review have not found significant improvements in adiponectin and leptin levels after 12 or 24 weeks of combined exercise in sedentary adults^34,58,60,63^. Our study findings partially agree with those previously reported in a meta-analysis^39^, where exercise (different types) was not effective to improve adiponectin; however, exercise training was significantly associated with reduced levels of leptin in adults with and without diabetes and other comorbidities. Another review also did not find significant improvements in adiponectin concentrations and speculated as possible reasons the intervention length of exercise, the intensity, the initial degree of inflammation of the participants, blood collection time, the menstrual cycle of women, among others^96^. In this way, we consider that there is still not enough evidence about the effect of combined exercise on adiponectin and leptin levels in sedentary adults.

Moreover, the pairwise meta-analysis suggested that combined exercise programs could significantly reduce TNF-α and CRP levels in physically inactive adults. No significant effect was observed for IL-6 concentration. These results are relevant since TNF-α and CRP are important pro-inflammatory cytokine factors, and their levels are directly associated with adiposity and IR^94^. Consistent with our findings, a previous literature review discussing the effects of physical activity on inflammatory cytokine mediators also suggested that combined aerobic and resistance exercise could be effective in improving inflammatory state^97^. An RCT included in our meta-analysis^66^ found that 12-weeks of combined exercise training significantly reduced TNF-α levels in postmenopausal women with abdominal obesity. The authors^66^ supposed that this results from reduced adipose tissue secretion of TNF-α after combined exercise decreased body fat and visceral fat mass. Donges et al.^16^ also found that despite reduced abdominal fat mass and plasma IL-6 and TNF-α concentrations after 12-weeks of exercise, no corresponding effects on CRP concentration were evident, owing to the prospect that concentrations of CRP were not elevated to a great enough extent at baseline to warrant a reduction after the exercise program. It would be expected that a reduction of IL-6 and TNF-α, as systemic drivers of CRP synthesis and release, would liken a reduction of basal CRP concentrations and thus reduce prospective T2DM and cardiovascular diseases risk^16^. On the other hand, Brunelli et al.^32^ despite not have found significant reductions in TNF-α and IL-6 concentrations, a significant reduction of approximately 118% in CRP levels was observed after 24-weeks of exercise program. The authors remarked that the participants with elevated CRP levels at the baseline may show major changes in CRP after the training period. Furthermore, this study also found that the changes in CRP were positively associated with the changes in the percentage of body fat for combined training group.

These findings are also consistent with a meta-analysis that verified that exercise training reduces CRP levels, and that the magnitude of the effect is higher when exercise is accompanied by a decrease in BMI or fat mass, which suggests the importance of improving body composition to reduce inflammatory factors^98^. Pérez-López et al.^33^ found that 12 weeks of combined training caused differences in circulating concentrations of four myokines that are implicated in glucose and lipid metabolism, including a reduction of 16% in IL-6 levels in sedentary postmenopausal women with obesity. The reduction of these circulating myokines was inversely associated with fat-free mass; these results could be attributed to a resistance exercise-induced inflammation to facilitate muscle cell repair and the exercise adaptations associated with the hypertrophic process^33^. According to Streb et al.^37^, the levels of inflammatory markers evaluated were unaltered, possibly due to the acute effect of exercise training and the current state of health of the participants.

These results should be interpreted with caution due to the small number of studies included in the analysis of these outcomes, and because few studies reported the time elapsed between the last exercise session and plasma/serum measurement. The time of blood draws plays an important role in cytokine marker analysis, due to the distinct acute and chronic effects of exercise. It is well known that exercise exerts a cascade of inflammatory events which leads to long-term anti-inflammatory effects on health^99^. A single bout of exercise initiates a complex and time-dependent cascade of inflammatory events which depends, among other factors, of exercise intensity and duration, and individuals exercise capacity of exercise^99^. The inflammatory cascade induced by acute exercise in skeletal muscle is characterized by an initial pro-inflammatory response (approximately 1.5–24 h post exercise) followed by an anti-inflammatory muscle regenerative response (approximately 24–72 h post exercise)^99^. In addition, acute exercise initiates inflammatory processes in the systemic circulation with significant releases of circulating myokines (i.e., IL-6) which is followed by a subsequent release of circulating anti-inflammatory cytokines (i.e., IL-10 and IL-1ra)^11,99^. Whereas acute bouts of exercise transiently increase inflammation, chronic adaptation to exercise training has an anti-inflammatory effect that may be mediated via both the reduction in visceral adipose tissue (with subsequent decreased release of adipokines) and the induction of an anti-inflammatory environment with each bout of exercise^11,24^. Of the 12 studies included in this meta-analysis that assessed at least one cytokine marker, 5 reported the time of blood draw. In two studies^32,62^, blood was collected 72 h after the last exercise session. In another study^37^, blood was collected 48 and 72 h after the last exercise session. Moreover, Libardi et al.^38^ and Mendez-Gutierrez et al.^58^ reported that blood collections were done 7 days and 4–5 days after the training program, respectively. No details about blood collection time were presented by the other studies.

Evidence suggests that combined exercise could reduce the inflammatory cytokines levels through several mechanisms. Combined exercise training may reduce the secretion of inflammatory cytokines by reducing % body fat mass, mainly visceral fat mass. As we mentioned previously, adipose tissue secretes a wide variety of inflammatory cytokines, and some of the current RCTs included in this review, and previous findings^96^ have shown that combined training is effective to reduce % of body fat mass and abdominal fat mass. Furthermore, combined training promotes the production of skeletal muscle mass, improves muscle protein synthesis, and preserves myocellular quality despite weight loss^100^. Moreover, as previously described, muscle-derived IL-6 increased with exercise, appears to have direct anti-inflammatory effects (inhibiting TNF-α production and stimulating the incidence of anti-inflammatory cytokines like IL-1ra and IL-10), and functions as a mechanism to enhance glucose tolerance^11^. In addition, exercise training may reduce chronic inflammation by reducing inflammatory cytokine production by endothelial cells and innate immune cells^101^.

We also attempted to analyze other pro- and anti-inflammatory cytokines such as IL-1β, MCP-1, IL-15, IL-10, IL-1ra, among others. However, we did not find enough literature on these cytokines, so it was not possible to conduct a meta-analysis. Nevertheless, 4 of the RCTs included in this review showed data for IL-15^32,33^, IL-1β^62^, or IL-1ra^16^. IL-1β is known as a key pro-inflammatory mediator of β-cell damage in T2DM^11^. The study of Salamat et al.^62^ found that 8-weeks of combined exercise promoted a significant decrease in IL-1β concentration in overweight men. IL-15 is an anabolic factor that is highly expressed in the skeletal muscle and appears to be involved in the long-term regulation of abdominal obesity^11^. A study of Brunelli et al.^32^ observed an increase in serum concentrations of IL-15 after 24 weeks of combined exercise in middle-aged adults. According to the authors, the increase in fat-free-mass and decreases in body fat mass observed in the exercise group may have contributed to the increases in serum IL-15 concentrations, and consequently, higher levels of IL-15 may have influenced the improvements in IR parameters. Contradictory results were observed in the study of Pérez-López et al.^33^, where authors found a significant reduction of serum IL-15 levels (-31%) after 12-weeks of combined exercise. The anti-inflammatory IL-1ra operates by suppressing the effects of IL-1^93^. Interestingly, obese individuals present higher serum levels of IL-1ra that are associated with increased BMI and IR, as reviewed elsewhere^93^. The study of Donges et al.^16^ revealed no effect of training on IL-1ra concentrations. More studies are needed to analyze the impact of combined training on these cytokines.

### Study limitations

To our knowledge, no meta-analysis has analyzed the effect of the combined training program on glucose metabolism markers and inflammatory cytokine levels in sedentary adults without diabetes, which seems to be an important public health issue. A major strength of our study was the inclusion of RCT studies with only an exercise component, not being affected by the influence of other common approaches like diet caloric restriction. However, we recognize that there are 6 limitations to this study that should be considered. First, our search strategy only included studies with defined terms in English on the title from specifically selected electronic databases; this could potentially overlook other relevant publications written in other languages^44^. Second, the oral glucose tolerance test was not included in this review as one of the measures for glucose metabolism. The inclusion of this outcome would have been important to understand if combined training improves glucose tolerance. Third, considerable unexplained heterogeneity was detected for all outcomes, except for TNF-α and CRP. In relation to the HOMA-IR index, we performed meta-regression models considering the age, sex, and intervention length as potential explanatory variables; however, these models were not able to explain any variation in the effect size. The heterogeneity detected in the different outcomes could be partially justified by potential differences in methodology (method of collecting blood samples, time elapsed between last exercise session and cytokines measurement, applied procedures and instruments to determine biomarkers), participants’ characteristics (i.e., BMI, menopausal status, baseline level of glucose or inflammation), exercise program protocol (i.e., differences in intensity and volume of aerobic/resistance training, exercise equipment, and order of aerobic/ resistance exercise). Furthermore, some of the main outcomes have few studies included, and some of them have small sample sizes, which limits the statistical power and prevents us from establishing robust conclusions. Small samples may contribute to small sample bias^102^. The significant heterogeneity found in the outcomes generates uncertainty about the real effects of combined training in these biomarkers and should therefore be interpreted with caution. Fourth, there was evidence for significant publication bias in Egger’s test for fasting glucose, insulin, and HOMA-IR analyses. Sixthly, we did not consider, in our subgroup or meta-regression analysis, the sequence of performing aerobic and resistance exercises because some of the included studies used a mixed circuit training methodology, performed aerobic and resistance training on separate days, or did not specify the order/sequence. However, the evidence on this topic is scarce. We found one study^103^ that assessed the sequence of combined training (i.e., resistance plus aerobic training vs. aerobic plus resistance training) within a multi-professional context focusing on the treatment of obesity with a cohort of adolescents. The authors found that regardless of the order in which combined exercise was performed, body composition, insulin, HOMA-IR, triglycerides, total cholesterol, and LDL-C were significantly improved.

## Conclusions

This systematic review and meta-analysis allow us to conclude that combined exercise training can be an effective strategy to improve some glucose metabolism markers, such as fasting glucose, insulin, and HOMA-IR in sedentary adults without diabetes. Additionally, significant improvements in TNF-α and CRP were also observed in favor of combined training, however, conclusions cannot be made as precisely due to the few studies found. No significant effects were observed for HbA1c, adiponectin, leptin, and IL-6. Thus, our study could have important clinical and public health implications supporting the importance of performing combined aerobic and resistance exercises to improve glucose metabolism, which could help prevent IR, T2DM, and its complications in sedentary adults. However, future studies are needed to better define the dose–response effects of combined exercise training on glucose metabolism markers and chronic inflammation and to further explore the molecular mechanisms by which combined exercise can effectively improve insulin sensitivity and the degree of inflammation. Furthermore, future meta-analysis should include studies that have both metabolic and inflammatory indicators so that a relationship between the two can be established.

### Supplementary Information

Supplementary Information.

## Data Availability

The datasets generated during and/or analyzed during the current study are available from the corresponding author on reasonable request.
