# Supplementary material for: The effects of combined exercise training on glucose metabolism and inflammatory markers in sedentary adults: a systematic review and meta-analysis
Source: Sci Rep. 2024 Jan 22;14:1936. doi: 10.1038/s41598-024-51832-y (PMC10803738; doi:10.1038/s41598-024-51832-y)
Supplement: Supplementary file 1 — Supplementary Information. [file 41598_2024_51832_MOESM1_ESM.docx]

**Supplementary material**

**The Effects of Combined Exercise Training on Glucose Metabolism and Inflammatory Markers in Sedentary Adults: A Systematic Review and Meta-Analysis**

Fernanda M. Silva^1,2*^, Pedro Duarte-Mendes^3,4^, Ana M. Teixeira^1,2^, Carlos M. Soares^1,2,5^, and José P. Ferreira^1,2^

**Affiliations:**

^1^ Faculty of Sport Sciences and Physical Education, FCDEF, University of Coimbra, Coimbra, Portugal; ^2^ Research Unit for Sport and Physical Activity (CIDAF, uid/dtp/04213/2020), University of Coimbra, Coimbra, Portugal; ^3^ Department of Sports and Well-being, Polytechnic Institute of Castelo Branco, Castelo Branco, Portugal; ^4^ Sport, Health & Exercise Research Unit (SHERU), Polytechnic Institute of Castelo Branco, Castelo Branco, Portugal; ^5^ Molecular Physical-Chemistry R & D Unit, Department of Chemistry, University of Coimbra, Coimbra, Portugal.

*** Corresponding Author:** Fernanda M. Silva

Faculty of Sport Sciences and Physical Education, University of Coimbra

Avenida de Conímbriga, Estádio Universitário de Coimbra, Pavilhão 3

3040-248 Coimbra, Portugal

E-mail: geral.fernandasilva@gmail.com

**Table S1** Search strategy for Scopus, Pubmed, Web of Science, and Cochrane Library

| *Database* | *Search strategy* |
| --- | --- |
| Scopus (3294) | TITLE ( ( ( adult* OR "healthy subjects" OR "healthy individuals" OR "sedentary subjects" OR "Middle Aged" OR aged OR old OR older OR worker* OR elderly OR man OR men OR women OR woman ) AND ( exercise* OR "physical activity" OR training OR "combined training" OR "combined aerobic and resistance" OR endurance OR strength OR "concurrent training" OR program* ) AND ( "insulin resistance" OR "insulin sensitivity" OR insulin OR glucose OR homa OR homa-ir OR glycemic OR glycaemic OR metabolism OR "metabolic syndrome" OR "metabolic health" OR "metabolic markers" OR "metabolic profile" OR "metabolic responses" OR "metabolic risk" OR "metabolic parameters" OR cardiometabolic OR hba1c OR "glycated hemoglobin" OR igf-1 OR leptin OR adiponectin OR adipocytokine* OR adipokine* OR cytokine* OR interleukin* OR inflammation OR inflammatory OR "anti-inflammatory" OR biomarker* OR marker* OR il-6 OR tnf-α OR "tumor necrosis factor-alpha" OR il-1β OR mcp-1 ) AND NOT ( animal* OR mice OR mouse OR rats OR child OR children OR adolescents OR pediatric ) ) ) AND ( LIMIT-TO ( DOCTYPE , "ar" ) OR LIMIT-TO ( DOCTYPE , "cp" ) OR LIMIT-TO ( DOCTYPE , "ch" ) ) |
| Web of Science (4361) | Citation Report: ((adult* OR “healthy subjects” OR “healthy individuals” OR “sedentary subjects” OR “Middle Aged” OR aged OR old OR older OR worker* OR elderly OR man OR men OR women OR woman) AND (exercise* OR “physical activity” OR training OR “combined training” OR “combined aerobic and resistance” OR endurance OR strength OR “concurrent training” OR program*) AND (“insulin resistance” OR “insulin sensitivity” OR insulin OR glucose OR HOMA OR HOMA-IR OR glycemic OR glycaemic OR metabolism OR “metabolic syndrome” OR “metabolic health” OR “metabolic markers” OR “metabolic profile” OR “metabolic responses” OR “metabolic risk” OR “metabolic parameters” OR cardiometabolic OR HbA1c OR “glycated hemoglobin” OR IGF-1 OR leptin OR adiponectin OR adipocytokine* OR adipokine* OR cytokine* OR interleukin* OR inflammation OR inflammatory OR “anti-inflammatory” OR biomarker* OR marker* OR IL-6 OR TNF-α OR “tumor necrosis factor-alpha” OR IL-1β OR MCP-1) NOT (animal* OR mice OR mouse OR rats OR child OR children OR adolescents OR pediatric)) (Title) and Article or Meeting Abstract or Proceeding Paper or Book Chapters or Early Access (Document Types) |
| PubMed (1010) | ((adult*[Title] OR "healthy subjects"[Title] OR "healthy individuals"[Title] OR "sedentary subjects"[Title] OR "Middle Aged"[Title] OR aged[Title] OR old[Title] OR older[Title] OR worker*[Title] OR elderly[Title] OR man[Title] OR men[Title] OR women[Title] OR woman[Title]) AND (exercise*[Title] OR "physical activity"[Title] OR training[Title] OR "combined training"[Title] OR "combined aerobic and resistance"[Title] OR endurance[Title] OR strength[Title] OR "concurrent training"[Title] OR program*[Title]) AND ("insulin resistance"[Title] OR "insulin sensitivity"[Title] OR insulin[Title] OR glucose[Title] OR HOMA[Title] OR HOMA-IR[Title] OR glycemic[Title] OR glycaemic[Title] OR metabolism[Title] OR "metabolic syndrome"[Title] OR "metabolic health"[Title] OR "metabolic markers"[Title] OR "metabolic profile"[Title] OR "metabolic responses"[Title] OR "metabolic risk"[Title] OR "metabolic parameters"[Title] OR cardiometabolic[Title] OR HbA1c[Title] OR "glycated hemoglobin"[Title] OR IGF-1[Title] OR leptin[Title] OR adiponectin[Title] OR adipocytokine*[Title] OR adipokine*[Title] OR cytokine*[Title] OR interleukin*[Title] OR inflammation[Title] OR inflammatory[Title] OR "anti-inflammatory"[Title] OR biomarker*[Title] OR marker*[Title] OR IL-6[Title] OR TNF-α[Title] OR "tumor necrosis factor-alpha"[Title] OR IL-1β[Title] OR MCP-1[Title]) NOT (animal*[Title] OR mice[Title] OR mouse[Title] OR rats[Title] OR child[Title] OR children[Title] OR adolescents[Title] OR pediatric[Title])) |
| Cochrane  (2031) | Trials matching ((adult* OR “healthy subjects” OR “healthy individuals” OR “sedentary subjects” OR “Middle Aged” OR aged OR old OR older OR worker* OR elderly OR man OR men OR women OR woman) AND (exercise* OR “physical activity” OR training OR “combined training” OR “combined aerobic and resistance” OR endurance OR strength OR “concurrent training” OR program*) AND (“insulin resistance” OR “insulin sensitivity” OR insulin OR glucose OR HOMA OR HOMA-IR OR glycemic OR glycaemic OR metabolism OR “metabolic syndrome” OR “metabolic health” OR “metabolic markers” OR “metabolic profile” OR “metabolic responses” OR “metabolic risk” OR “metabolic parameters” OR cardiometabolic OR HbA1c OR “glycated hemoglobin” OR IGF-1 OR leptin OR adiponectin OR adipocytokine* OR adipokine* OR cytokine* OR interleukin* OR inflammation OR inflammatory OR “anti-inflammatory” OR biomarker* OR marker* OR IL-6 OR TNF-α OR “tumor necrosis factor-alpha” OR IL-1β OR MCP-1) NOT (animal* OR mice OR mouse OR rats OR child OR children OR adolescents OR pediatric)) in Record Title |

| **Author (year)** | **Group** | ***n*** | **BMI (kg/m2)** | | | **Waist circumference** | | | **Fat mass (kg)** | | | **Body fat mass (%)** | | | **Skeletal muscle mass (kg)** | | | **VO_2_max** | | | |
| --- | --- | --- | --- | --- | --- | --- | --- | --- | --- | --- | --- | --- | --- | --- | --- | --- | --- | --- | --- | --- | --- |
|  |  |  | **Pre** | **Post** | ***p*** | **Pre** | **Post** | ***p*** | **Pre** | **Post** | ***p*** | **Pre** | **Post** | ***p*** | **Pre** | **Post** | ***p*** | **Pre** | **Post** | ***p*** |  |
|  |  |  | **Mean ± SD or (SE)** | **Mean ± SD or (SE)** |  | **Mean ± SD or (SE)** | **Mean ± SD or (SE)** |  | **Mean ± SD or (SE)** | **Mean ± SD or (SE)** |  | **Mean ± SD or (SE)** | **Mean ± SD or (SE)** |  | **Mean ± SD or (SE)** | **Mean ± SD or (SE)** |  | **Mean ± SD or (SE)** | **Mean ± SD or (SE)** |  |  |
| Silva et al. (2022) | Control | 19 | 28.75 ± 4.3 | 28.97 ± 4.7 | > 0.05 | 95.59 ± 11.3 | 97.88 ± 11.7 | **0.03** | 28.13 ± 8.8 | 27.95 ± 9.5 | > 0.05 | 37.71 ± 7.4 | 37.05 ± 7.9 | > 0.05 | 25.30 ± 6.3 | 25.71 ± 6.53 | > 0.05 |  |  |  |  |
|  | Exercise | 12 | 26.37 ± 3.9 | 26.31 ± 3.9 | > 0.05 | 91.32 ± 13.1 | 88.90 ± 12.7 | **0.01** | 24.24 ± 9.0 | 23.98 ± 8.9 | > 0.05 | 34.42 ± 7.9 | 34.13 ± 7.9 | > 0.05 | 24.95 ± 5.4 | 24.97 ± 5.46 | > 0.05 |  |  |  |  |
| Amaro-Gahete et al. (2021) | Control | 6 | 32.5 ± 3.0 | 33.2 ± 3.3 | > 0.05 | 108.0 ± 6.1 | 107.9 ± 8.0 | > 0.05 | 28.5 ± 7.3 | 30.7 ± 7.6 | > 0.05 |  |  |  | 69.3 ± 6.9 | 69.3 ± 7.4 | > 0.05 | 25.6 ± 2.3 | 27.7 ± 2.6 | > 0.05 |  |
|  | Exercise | 6 | 32.1 ± 3.6 | 31.4 ± 2.7 | **< 0.05** | 105.4 ± 9.4 | 104.1 ± 8.3 | > 0.05 | 27.8 ± 6.6 | 26.6 ± 4.8 | **< 0.05** |  |  |  | 66.3 ± 9.3 | 65.3 ± 8.4 | > 0.05 | 27.4 ± 7.4 | 30.7 ± 5.5 | > 0.05 |  |
| Amaro-Gahete et al. (2019) | Control | 17 | 26.39 ± 3.8 | 26.21 ± 3.7 | NA | 92.46 ± 10.8 | 92.30 ± 11.6 | NA |  |  |  |  |  |  |  |  |  |  |  |  |  |
|  | Exercise | 17 | 25.41 ± 2.9 | 24.90 ± 2.4 | NA | 90.43 ± 11.0 | 88.54 ± 10.2 | NA |  |  |  |  |  |  |  |  |  |  |  |  |  |
| Brunelli et al. (2015) | Control | 13 | 31.01 ± 0.4 | 31.09 ± 0.5 | > 0.05 | 101.82 ± 1.3 | 102.49 ± 1.4 | > 0.05 |  |  |  | 32.35 ± 1.8 | 31.10 ± 1.8 | > 0.05 |  |  |  | 29.05 ± 1.2 | 29.02 ± 1.1 | > 0.05 |  |
|  | Exercise | 17 | 30.95 ± 0.4 | 30.65 ± 0.4 | > 0.05 | 103.04 ± 1.3 | 100.74 ± 1.4 | > 0.05 |  |  |  | 35.96 ± 1.4 | 28.59 ± 1.6 | **< 0.05** |  |  |  | 28.02 ± 1 | 31.09 ± 1 | **< 0.05** |  |
| Bonfante et al. (2017) | Control | 12 | 30.87 ± 1.8 | 30.53 ± 1.6 | > 0.05 | 101.41 ± 4.2 | 103.26 ± 5.0 | > 0.05 |  |  |  | 32.71 ± 5.4 | 35.54 ± 6.3 | > 0.05 |  |  |  | 27.17 ± 4.7 | 27.61 ± 3.9 | > 0.05 |  |
|  | Exercise | 10 | 30.86 ± 1.5 | 31.15 ± 1.9 | > 0.05 | 102.32 ± 5.3 | 99.62 ± 4.9 | **< 0.05** |  |  |  | 35.25 ± 5.2 | 28.57 ± 6.9 | **< 0.05** |  |  |  | 26.81 ± 3.6 | 29.87 ± 3.9 | **< 0.05** |  |
| Donges et al. (2013) | Control | 8 |  |  |  |  |  |  | 23.2 (3.8) | 23.9 (4.1) | > 0.05 | 23.9 (2.2) | 24.4 (2.3) | > 0.05 |  |  |  |  |  |  |  |
|  | Exercise | 13 |  |  |  |  |  |  | 23.6 (1.4) | 22.2 (1.5) | **< 0.05** | 24 (1.2) | 22.6 (1.3) | **< 0.05** |  |  |  |  |  |  |  |
| Libardi et al. (2012) | Control | 13 | 24.7 ± 3.3 | 24.5 ± 3.3 | > 0.05 | 85.5 ± 9.7 | 85.8 ± 10.1 | > 0.05 |  |  |  |  |  |  |  |  |  | 30.9 ± 5.4 | 31.10 ± 4.4 | > 0.05 |  |
|  | Exercise | 11 | 28.37 ± 3.0 | 28.18 ± 3.2 | > 0.05 | 94.7 ± 5.6 | 92.69 ± 5.5 | > 0.05 |  |  |  |  |  |  |  |  |  | 31.18 ± 5.5 | 34.43 ± 5.4 | **< 0.05** |  |
| Rahimi et al. (2020) | Control | 10 |  |  |  | 109.7 ± 8.6 | 108.38 ± 7.9 | > 0.05 |  |  |  | 42.8 ± 4.7 | 43.17 ± 5.1 | > 0.05 | 37.27 ± 2.8 | 37.29 ± 2.8 | > 0.05 | 26 ± 4.7 | 25.31 ± 4.1 | > 0.05 |  |
|  | Exercise | 10 |  |  |  | 106.4 ± 8.5 | 87.65 ± 6.4 | **< 0.05** |  |  |  | 43 ± 5.2 | 30.28 ± 5.1 | **< 0.05** | 36.58 ± 3.8 | 38.86 ± 4 | **< 0.05** | 27.1 ± 3.6 | 35.16 ± 5.5 | **< 0.05** |  |
| Sillanpää et al. (2009) | Control | 12 |  |  |  |  |  |  |  |  |  | 32.6 ± 6.8 | 32.0 ± 1.5 | > 0.05 |  |  |  |  |  |  |  |
|  | Exercise | 18 |  |  |  |  |  |  |  |  |  | 30.9 ± 6.1 | 29.0 ± 1.7 | **< 0.01** |  |  |  |  |  |  |  |
| Pérez-López et al. (2021) | Control | 12 | 34.9 ± 6.4 | 36.9 ± 6.6 | > 0.05 |  |  |  | 44.1 ± 10 | 44 ± 9.5 | > 0.05 |  |  |  |  |  |  |  |  |  |  |
|  | Exercise | 13 | 33.8 ± 5.3 | 33.3 ± 5.5 | > 0.05 |  |  |  | 38 ± 8.1 | 36.6 ± 8.5 | **< 0.05** |  |  |  |  |  |  |  |  |  |  |
| Shabani et al. (2019) | Control | 10 | 28.6 ± 2.4 | 28.8 ± 2.5 | **0.003** |  |  |  |  |  |  |  |  |  |  |  |  | 20.3 ± 2.0 | 20.4 ± 2.3 | 0.7 |  |
|  | Exercise | 12 | 28.4 ± 1.1 | 27.8 ± 1.2 | **0.001** |  |  |  |  |  |  |  |  |  |  |  |  | 19.5 ± 0.9 | 23.2 ± 2.2 | **0.001** |  |
| Azarbayjani et al. (2014) | Control | 10 | 24 ± 0.8 | 24.1 ± 0.8 | > 0.05 |  |  |  |  |  |  | 18.7 ± 1.2 | 18.8 ± 1.2 | > 0.05 |  |  |  | 35.7 ± 2.5 | 35.9 ± 2.3 | > 0.05 |  |
|  | Exercise | 10 | 23.3 ± 1 | 23.3 ± 1 | > 0.05 |  |  |  |  |  |  | 18.11 ± 1.3 | 16 ± 0.4 | **< 0.05** |  |  |  | 36.3 ± 3.8 | 40.3 ± 3.2 | **< 0.05** |  |
| Ha et al. (2015) | Control | 9 | 24 ± 1.6 | 24.32 ± 2.2 | NA |  |  |  |  |  |  | 33.11 ± 1.8 | 33.67 ± 1.9 | NA | 38.03 ± 4.4 | 37.85 ± 4.4 | NA |  |  |  |  |
|  | Exercise | 9 | 24.71 ± 2.5 | 24.39 ± 2.8 | NA |  |  |  |  |  |  | 33.92 ± 2.6 | 32.33 ± 3.4 | **< 0.05** | 41.37 ± 7.1 | 41.72 ± 7.1 | NA |  |  |  |  |
| Park et al. (2015) | Control | 10 |  |  |  |  |  |  |  |  |  | 34.6 ± 1.6 | 34.8 ± 1.7 | > 0.05 | 41.2 ± 3.5 | 40.7 ± 3.4 | > 0.05 | 27.67 ± 3.7 | 26.89 ± 3.4 | > 0.05 |  |
|  | Exercise | 10 |  |  |  |  |  |  |  |  |  | 34.3 ± 2.8 | 31.4 ± 2.6 | **< 0.05** | 41 ± 2.83 | 42.9 ± 2.5 | > 0.05 | 27.16 ± 3.4 | 31.75 ± 3.9 | **< 0.01** |  |
| Asad et al. (2012) | Control | 10 | 29.26 ± 4.3 | 29.29 ± 4.4 | NA |  |  |  |  |  |  |  |  |  |  |  |  | 31.19 ± 6.4 | 31.02 ± 5 | NA |  |
|  | Exercise | 13 | 28.64 ± 3.8 | 27.38 ± 3.7 | NA |  |  |  |  |  |  |  |  |  |  |  |  | 27.83 ± 3.8 | 35.64 ± 6.4 | NA |  |
| Hara et al. (2005) | Control | 7 | 33.5 ± 5.6 | 32.6 ± 5.8 | > 0.05 |  |  |  | 28.6 ± 9.1 | 29.3 ± 8.2 | > 0.05 | 29.3 ± 3.8 | 30.3 ± 3 | > 0.05 | 30.3 ± 7.3 | 30.5 ± 6.3 | > 0.05 | 28.4 ± 4.4 | 24.9 ± 4.4 | > 0.05 |  |
|  | Exercise | 7 | 29.9 ± 3.8 | 28.6 ± 2.8 | **< 0.05** |  |  |  | 22.4 ± 5.5 | 18.6 ± 3.8 | **< 0.05** | 24.5 ± 3.6 | 21.3 ± 2 | **< 0.05** | 32.5 ± 5.7 | 32.5 ± 6.5 | > 0.05 | 30 ± 4.6 | 40.5 ± 1.6 | **< 0.05** |  |
| Rossi et al. (2017) | Control | 8 |  |  |  |  |  |  | 24.8 ± 7.6 | 25.2 ± 8.9 | NA | 40.4 ± 6.7 | 40.5 ± 7.4 | NA | 33.6 ± 3.6 | 33.6 ± 3.8 | > 0.05 |  |  |  |  |
|  | Exercise | 15 |  |  |  |  |  |  | 24.0 ± 4.8 | 23.3 ± 5.6 | NA | 39.4 ± 4.1 | 37.9 ± 4.8 | NA | 34.4 ± 4.1 | 35.4 ± 4.3 | > 0.05 |  |  |  |  |
| Álvarez et al. (2019) | Control | 14 | 32.0 ± 5.8 | 32.1 ± 6.0 | > 0.05 |  |  |  |  |  |  |  |  |  |  |  |  |  |  |  |  |
|  | Exercise | 14 | 29.8 ± 3.9 | 29.6 ± 3.5 | > 0.05 |  |  |  |  |  |  |  |  |  |  |  |  |  |  |  |  |

**Table S2** Changes on the body composition variables and VO_2max_ in the groups after the intervention

Note: Data pre and post not available in the studies of Streb et al. (2021), Streb et al. (2022), Mendez-Gutierrez et al. (2022), Gonçalves et al. (2021), Salamat et al. (2016), and Álvarez et al. (2021).


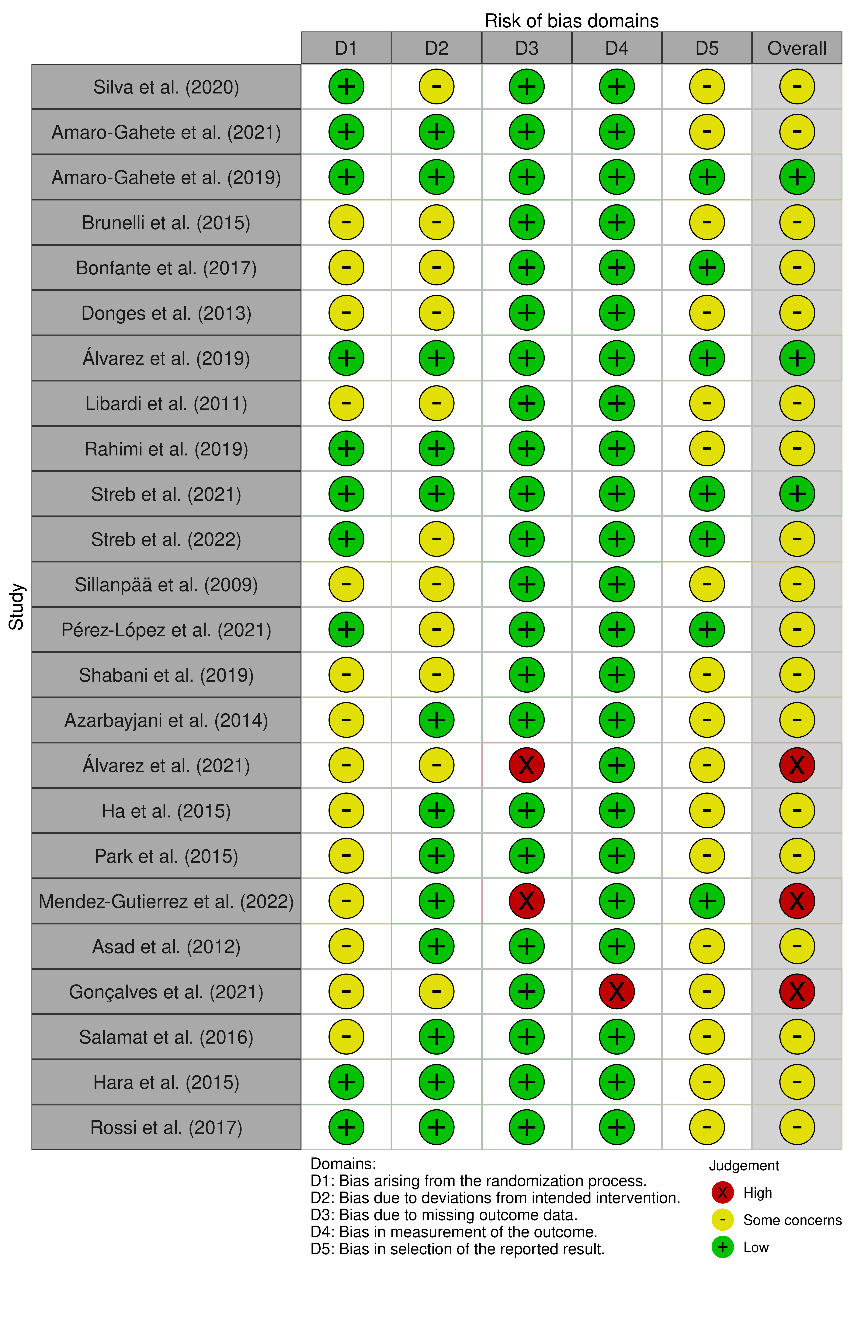


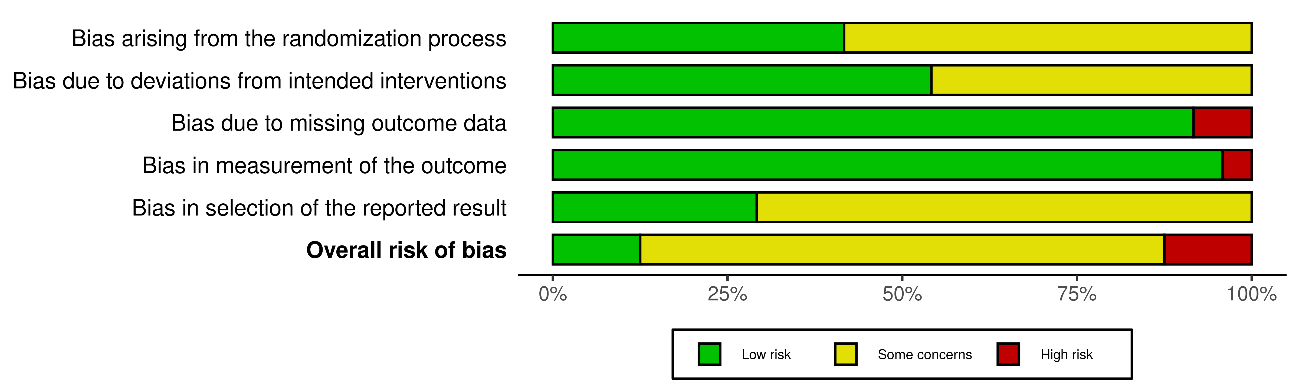


**Fig. S1** Risk-of-bias judgements using the RoB 2 tool


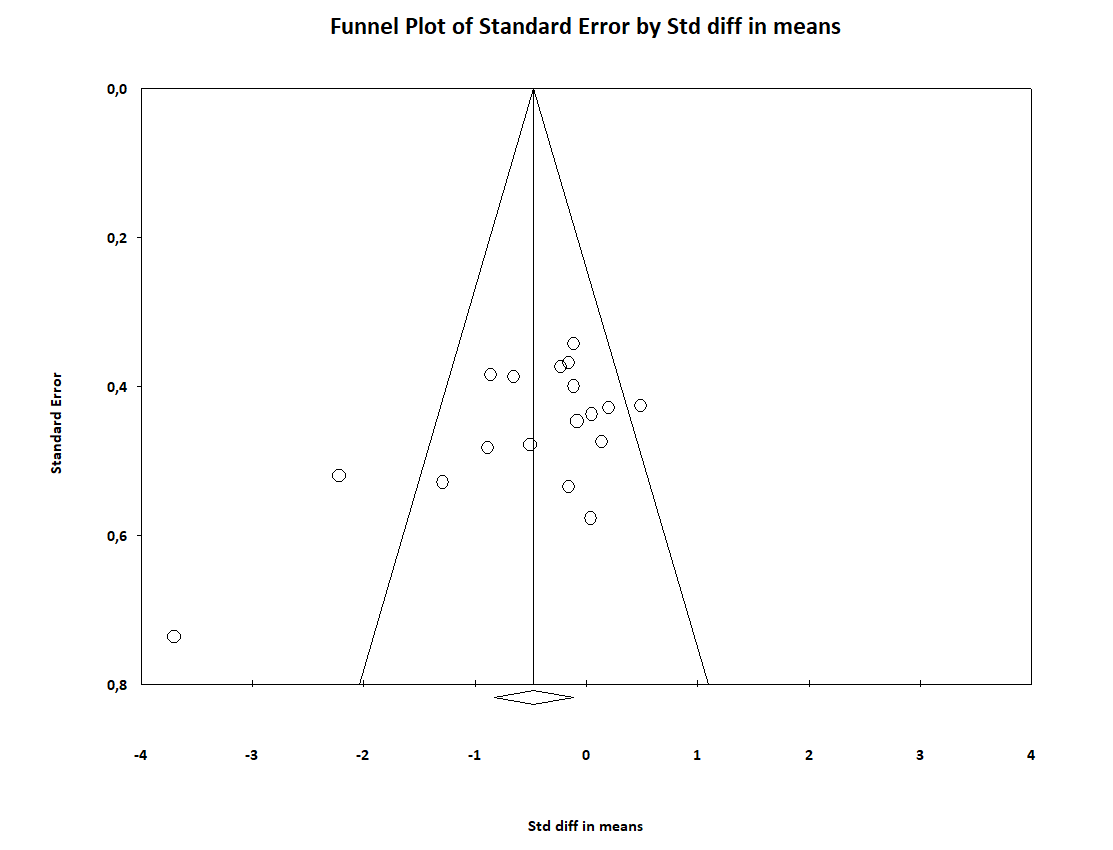


**Fig. S2** Funnel plot of standard error by std diff in means for fasting glucose


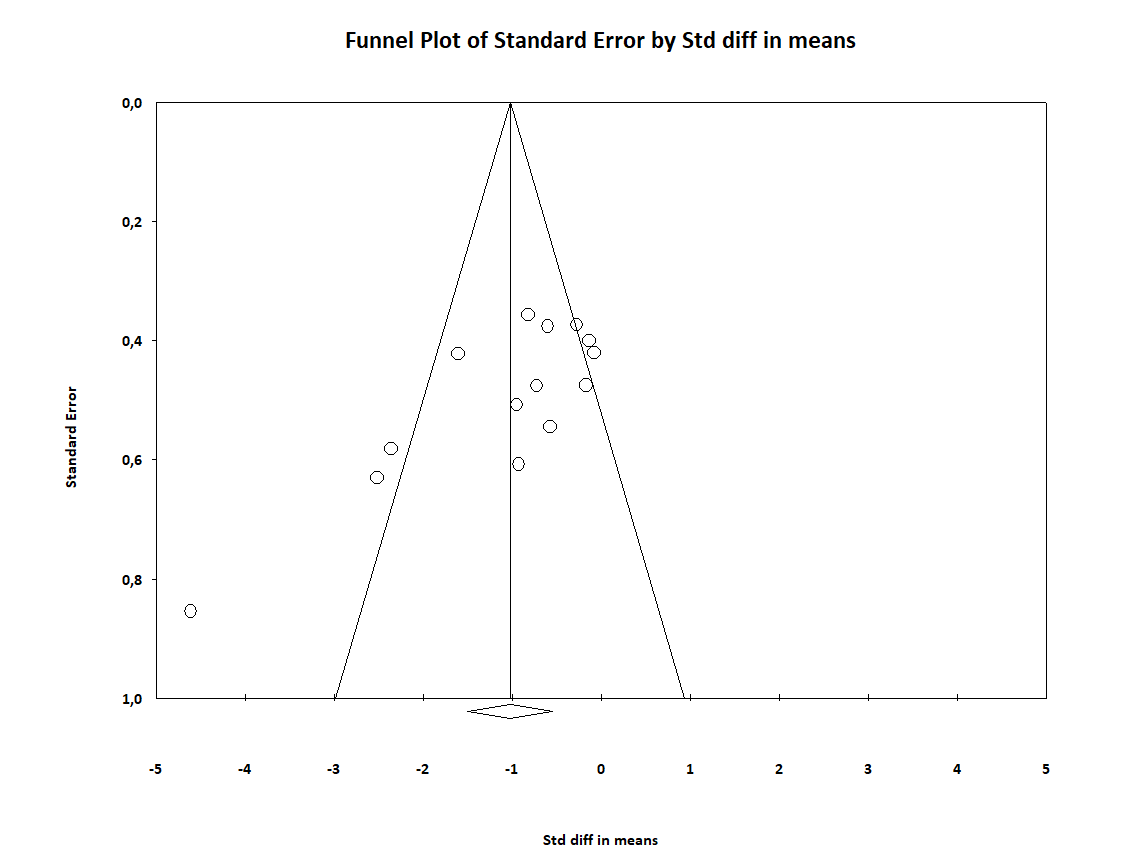


**Fig. S3** Funnel plot of standard error by std diff in means for fasting insulin


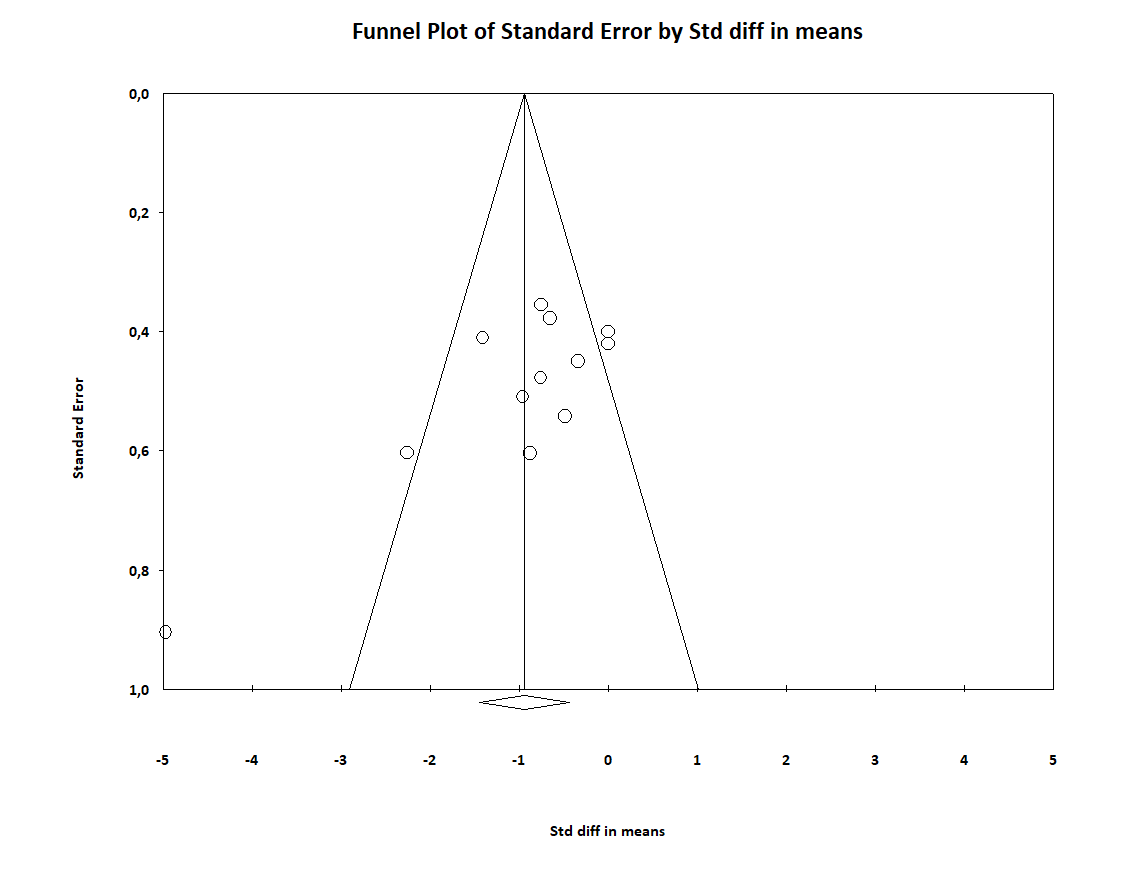


**Fig. S4** Funnel plot of standard error by std diff in means for HOMA-IR index

**Meta-Regression**

For studies that included HOMA-IR index, random effects meta-regression models were performed to examine whether three different covariates (mean age of the participants – ranging from 18.9 to 57.8 years old), sex – both sexes, women, and men, and intervention length – ranging from 12 to 24 weeks) influenced heterogeneity. We used regression to study the relationship between these covariates and effect size. Meta-regression was not performed for inflammatory outcomes that presented significant heterogeneity because this method should not be considered when there are fewer that ten studies in a meta-analysis.

Outcome: **HOMA-IR index**

Covariate: **sex (men, women, or both sexes included in studies)**

| **Main results for Model 1, Random effects (MM), Z-Distribution, Std diff in means** | | | | | | |
| --- | --- | --- | --- | --- | --- | --- |
| **Set** | **Coefficient** | **SE** | **95% Lower** | **95% Upper** | **Z-value** | ***P-value*** |
| Intercept | -0.7741 | 0.4558 | -1.6675 | 0.1192 | -1.7 | 0.0894 |
| Men | -0.5674 | 0.6342 | -1.8104 | 0.6756 | -0.89 | 0.371 |
| Women | 0.1254 | 0.7056 | -1.2576 | 1.5083 | 0.18 | 0.859 |

Notes: SE, standard error. Significant *p* was set at < 0.05. “Both sexes” was included as reference subgroup.

| **Test of the model: Simultaneous test that all coefficients (excluding intercept) are zero** |
| --- |
| Q = 1.25, df = 2, *p* = 0.5356 |
| **Goodness of fit: Test that unexplained variance is zero** |
| Tau² = 0.6457, Tau = 0.8036, I² = 74.15%, Q = 34.82, df = 9, *p* = 0.0001 |

The scatterplot presents the studies (circles), regression line, confidence interval and prediction interval.

**Summary:** The coefficient for men is -0.5674. This suggests that mean effect size in studies that only enrolled men was 0.567 lower than for studies which included both sexes (which represents greater magnitude of HOMA-IR reduction as result of intervention). The 95% confidence interval for the coefficient extends from -1.8104 to 0.6756. Similarly, the Z-value is -0.89 and the corresponding *p*-value is 0.371. The coefficient for women is 0.1254, which suggests that mean effect size in studies that only enrolled women was 0.1254 higher than for studies that include both sexes (which represents lower magnitude of HOMA-IR reduction as result of intervention). The 95% confidence interval extends from -1.2576 to 1.5083, the Z-value is 0.18, and corresponding p-value is 0.859. Therefore, we can accept the null hypothesis that the true value of the coefficient is zero. The Q-value for the set and the Q-value for the model are both 1.25 with *df* = 2 and corresponding *p*-value of 0.5356. The set model is not able to explain any of the variation in effect size. The τ^2^ as variance of true effects about the subgroup means is 0.6457, and the τ about the subgroup means is 0.8036. Further, the I^2^ statistic is 74.15% and the Q-value is 34.82 with 9 degrees of freedom and a corresponding *p*-value < 0.001. We conclude that the model does not fully explain the variation in effect size. Importantly, the relationship between sex and effect size is observational, not causal. Furthermore, these results should be interpreted with caution since the sex subgroups includes few studies (women: 3 studies; men = 5 studies; both sexes = 4 studies).

Outcome: **HOMA-IR index**

Covariate: **age (years)**

| **Main results for Model 1, Random effects (MM), Z-Distribution, Std diff in means** | | | | | | |
| --- | --- | --- | --- | --- | --- | --- |
|  | **Coefficient** | **SE** | **95% Lower** | **95% Upper** | **Z-value** | ***P-value*** |
| Intercept | -1.1401 | 0.898 | -2.9003 | 0.62 | -1.27 | 0.2042 |
| Age (years) | 0.0046 | 0.0216 | -0.0377 | 0.047 | 0.21 | 0.8298 |

Notes: SE, standard error. Significant *p* was set at < 0.05.

| **Test of the model: Simultaneous test that all coefficients (excluding intercept) are zero** |
| --- |
| Q = 0.05, df = 1, *p* = 0.8298 |
| **Goodness of fit: Test that unexplained variance is zero** |
| Tau² = 0.6169, Tau = 0.7854, I² = 73.77%, Q = 38.13, df = 10, *p* < 0.001 |

The scatterplot presents the studies (circles), regression line, confidence interval and prediction interval.

**Summary:** The coefficient age is 0.0046. The 95% confidence interval for the coefficient extends from -0.0377 to 0.047. Similarly, the Z-value is 0.21 and the corresponding *p*-value is 0.8298. Therefore, we can accept the null hypothesis that the true value of the coefficient is zero and conclude that there is no relationship between age and effect size. A test of the model yields a Q-value of 0.05 with 1 degree of freedom (df) and corresponding *p*-value of 0.8298. The model is not able to explain any of the variation in effect size. The variance of true effects about the regression line (represented by τ^2^) is 0.6169, and the standard deviation of true effects about the regression line is 0.7854 (τ). Further, the I^2^ statistic is 73.77% and the Q-value is 38.13 with 10 degrees of freedom and a corresponding *p*-value < 0.001. Thus, we can conclude that the model does not fully explain the variation in effect size. Importantly, the relationship between age and effect size is observational, not causal.

Outcome: **HOMA-IR index**

Covariate: **intervention length (number of weeks)**

| **Main results for Model 1, Random effects (MM), Z-Distribution, Std diff in means** | | | | | | |
| --- | --- | --- | --- | --- | --- | --- |
|  | **Coefficient** | **SE** | **95% Lower** | **95% Upper** | **Z-value** | ***P-value*** |
| Intercept | -1.0047 | 1.0131 | -2.9902 | 0.9809 | -0.99 | 0.3213 |
| Intervention length (weeks) | 0.0033 | 0.0654 | -0.1248 | 0.1315 | 0.05 | 0.9592 |

Notes: SE, standard error. Significant *p* was set at < 0.05.

| **Test of the model: Simultaneous test that all coefficients (excluding intercept) are zero** |
| --- |
| Q < 0.01, df = 1, *p* = 0.9592 |
| **Goodness of fit: Test that unexplained variance is zero** |
| Tau² = 0.6069, Tau = 0.7791, I² = 73.31%, Q = 37.46, df = 10, *p* < 0.001 |

The scatterplot presents the studies (circles), regression line, confidence interval and prediction interval.

**Summary:** The coefficient intervention length is 0.0033. The 95% confidence interval for the coefficient extends from -0.1248 to 0.1315. Similarly, the Z-value is 0.05 and the corresponding *p*-value is 0.9592. Therefore, we can accept the null hypothesis that the true value of the coefficient is zero and conclude that there is no relationship between intervention length and effect size. A test of the model yields a Q-value of < 0.001 with 1 degree of freedom and corresponding *p*-value of 0.9592. The model is not able to explain any of the variation in effect size. The τ^2^ is 0.6069, and the τ is 0.7791. Further, the I^2^ statistic is 73.31% and the Q-value is 37.46 with *df* = 10 and a corresponding *p*-value < 0.001. Thus, we can conclude that the model does not fully explain the variation in effect size. Importantly, the relationship between intervention length and effect size is observational, not causal.
